# Absorptivity Is an Important Determinant in the Toxicity Difference between Aristolochic Acid I and Aristolochic Acid II

**DOI:** 10.1021/acs.jafc.4c10765

**Published:** 2025-01-14

**Authors:** Hong-Ching Kwok, Hei-Tak Tse, Ka-Ki Ng, Shuangshuang Wang, Chun-Kit Au, Zongwei Cai, Wan Chan

**Affiliations:** † Department of Chemistry, The Hong Kong University of Science and Technology, Clear Water Bay, Kowloon, Hong Kong; ‡ Eastern Institute of Technology Ningbo, Ningbo, Zhejiang 315200, China; § Department of Chemistry and State Key Laboratory of Environmental and Biological Analysis, Hong Kong Baptist University, Kowloon Tong, Kowloon, Hong Kong SAR, China

**Keywords:** aristolochic acids, food contamination, dietary exposure, Balkan endemic nephropathy, DNA adduct

## Abstract

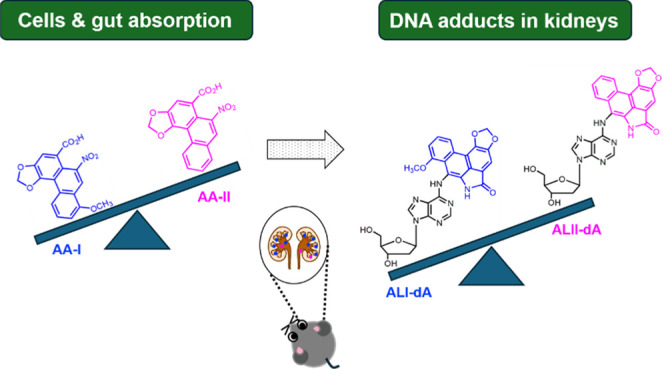

Inadvertent exposure to aristolochic acids (AAs) is causing chronic renal disease worldwide, with aristolochic acid I (AA-I) identified as the primary toxic agent. This study employed chemical methods to investigate the mechanisms underlying the nephrotoxicity and carcinogenicity of AA-I. Aristolochic acid II (AA-II), which has a structure similar to that of AA-I, was investigated with the same methods for comparison. Despite their structural similarities, findings from cultured human cells and gut sac experiments showed that AA-I is absorbed more effectively than AA-II (∼3 times greater for AA-I than for AA-II; *p* < 0.001). This increased absorption, along with the previously observed higher activity of reductive activation enzymes for AA-I, results in greater DNA damage and oxidative stress, both of which are key factors in AA-related toxicity. The similar patterns of cell mortality (34.4 ± 2.3% vs 9.7 ± 0.1% for AA-I and AA-II at 80 μM; *p* < 0.0001), DNA adduct formation (∼3 times greater for AA-I than for AA-II; *p* < 0.001), and oxidative stress levels in relation to the concentrations of AA-I and AA-II indicate that the higher absorption rate of AA-I is a significant contributor to its greater toxicity. The toxicity of AA-I was also found to be further enhanced by its (natural) coexistence with AA-II. Since AA-I and AA-II differ only by a methoxy group, future research on reducing risks associated with AA exposure should focus on strategies to lower the absorption of these compounds.

## Introduction

First identified in the 1920s, Balkan endemic nephropathy (BEN) is a slow progressive chronic tubulointerstitial disease primarily affecting residents of farming villages across various countries in the Balkan Peninsula.^1−3^ This disease is notable for its exclusivity to adults, with no reported cases in children; it is familial but not inherited, and it predominantly occurs in villages situated in low-lying valleys, closely associated with the development of carcinoma of the upper urothelial tract.^1,3−6^ The geographical confinement of BEN has prompted extensive collaborative research efforts involving scientists worldwide.^1,2,6−11^ However, despite decades of investigation, the precise etiology of BEN remains incompletely understood.^10−12^

**Scheme 1:**
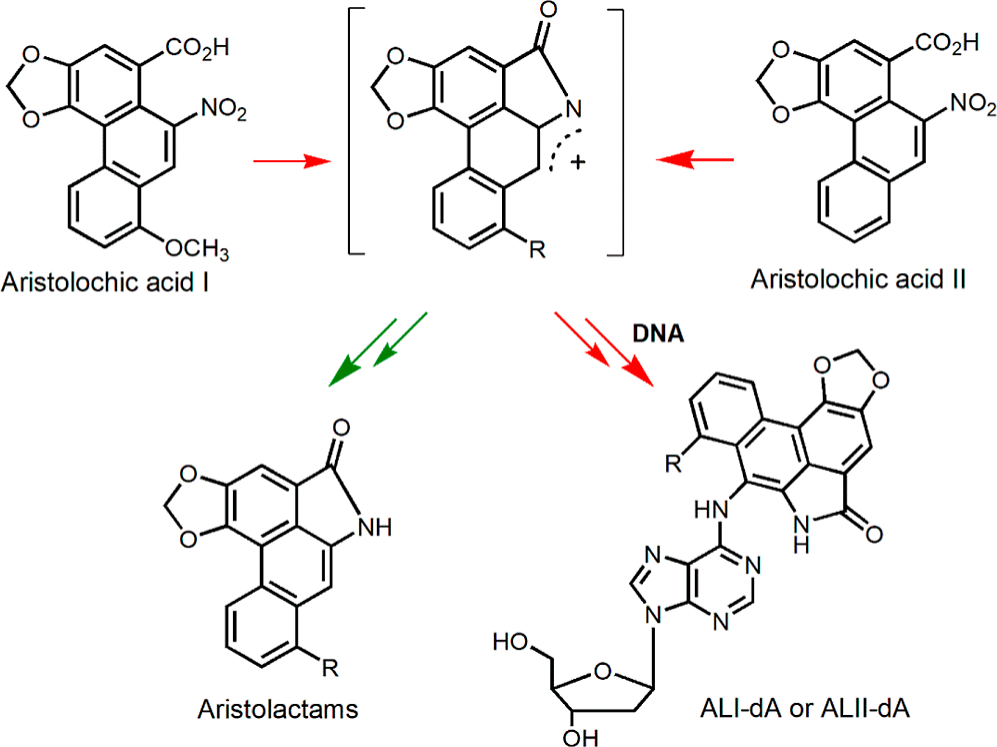
Metabolic Activation and DNA Adduct Formation of Aristolochic Acid I (R = OCH_3_) and Aristolochic Acid II (R = H)

Among various environmental factors, carcinogenic and nephrotoxic aristolochic acids (AAs; Scheme 1) have been suspected of contributing to the development of BEN since as early as 1970.^1,2,10−13^ Recently, the detection of AA-DNA adducts in the urothelial tissue of BEN patients had provided robust evidence linking the disease to environmental exposure to AAs.^1,5,11−14^ It is believed that the disease is triggered by prolonged consumption of AAs through bread made from flour derived from AA-contaminated wheat grains, potentially contaminated via two primary pathways. First, the seeds of *Aristolochia clematitis*, an AA-producing weed prevalent in the affected areas, were inadvertently harvested alongside wheat grains during machine harvesting.^1,15,16^ Second, AAs were absorbed by wheat plants through root uptake from AA-contaminated soil, subsequently accumulating in the wheat grains.^6,17−19^

**Scheme 2:**
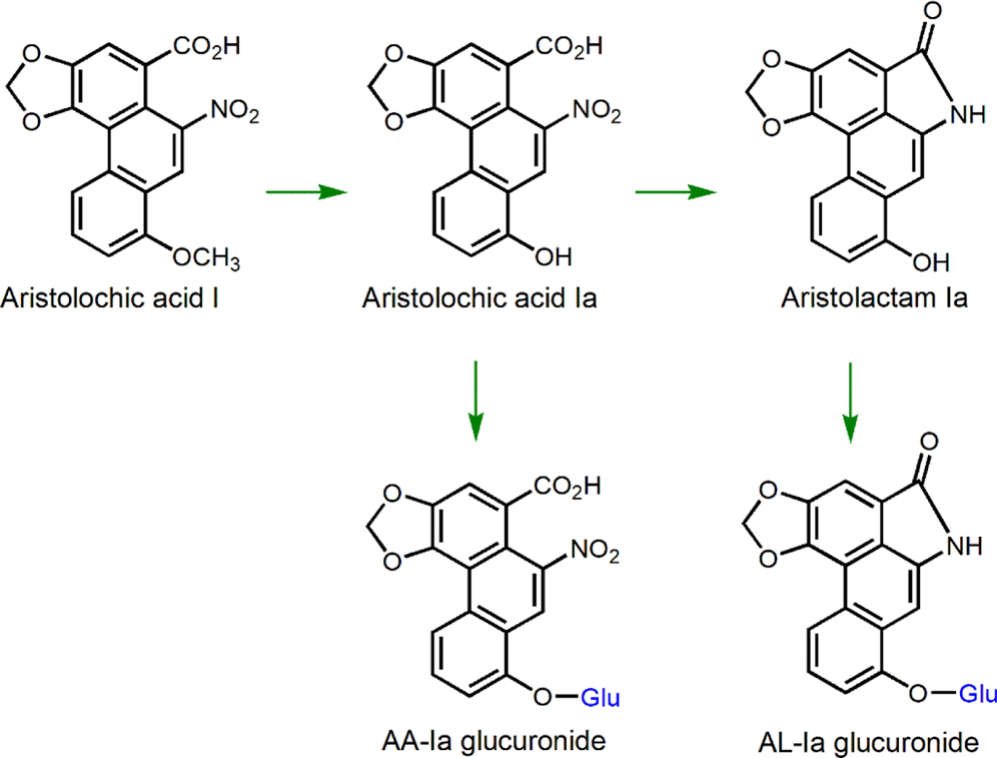
Metabolism of AA-I Forming AA-Ia and Aristolactam Ia, which Undergoes Glucuronide Conjugation, Lowering the Toxicity of AA-I

AAs are a family of structurally related nitrophenanthrene carboxylic acids (Scheme 1) found predominantly in the genus Aristolochiaceae, many of which have been used as herbal medicines.^14,20−22^ The major components of the AAs family are aristolochic acid I (AA-I) and aristolochic acid II (AA-II).^14,21,22^ The methoxy group that is only present on AA-I can be demethylated to form aristolochic acid Ia (AA-Ia), which is susceptible to aristolactam Ia formation and glucuronide conjugative detoxification (Scheme 2).^11,23,24^ On the contrary, previous studies have shown that AA-I is significantly more genotoxic than AA-II and is responsible for the majority of AA-induced carcinogenesis and nephrotoxicity.^25−30^ This difference had been attributed to the distinct enzyme activity of key enzymes responsible for the metabolic activation of AAs, such as NAD(P)H quinone dehydrogenase 1 (NQO1). For example, NQO1 was observed to be more efficient in reducing AA-I than AA-II.^31−34^ However, other factors that may also contribute to the differing toxicity of AA-I and AA-II, such as their relative absorptivity and the efficiency of their associated AA-DNA adduct excision from the genome, have remained largely unexplored.

Results from a previous toxicokinetic study revealed different rates of absorption, metabolic activation, detoxification, and elimination for AA-I and AA-II,^29^ shedding light on their potentially important, yet previously unrecognized, roles in the observed differences in toxicity between these compounds. Nevertheless, the conclusions were based on data from intraperitoneal injections of AA-I and AA-II mixtures, which differ from the common exposure route for humans—namely, oral ingestion through contaminated food or herbal decoctions. Furthermore, as demonstrated in the present study and in a study by Dedíková *et al.*, coexposure may affect the toxicokinetic of AA-I and/or AA-II.^35^

The goal of this study was to investigate the poorly understood mechanisms underlying the differing toxicity of AA-I and AA-II. Given that exposure-induced DNA damage is generally believed to be responsible for tumor development in both upper urinary tract and the renal fibrosis caused by AAs,^36−38^ we initially compared cell mortality rates and DNA damage in human kidney and liver cells exposed to either AA-I or AA-II. Specifically, we utilized a liquid chromatography–tandem mass spectrometry (LC–MS/MS) coupled with an isotope dilution method to quantitatively assess AA-DNA adducts (Scheme 1) and levels of oxidative stress-induced 8-oxo-2′-deoxyguanosine (8-oxo-dG) in cells subjected to varying durations and concentrations of AA exposure. Then, we determined cellular AAs, aristolactams (ALs), and glutathione (GSH) levels as indicators of absorptivity, metabolism, and oxidative stress in exposed cells, respectively. Additionally, we also investigated the effects of coexposure to AA-I and AA-II on cells and the rates at which AA-DNA adducts were formed and eliminated in the exposed cells. Finally, we quantified exposure-induced AA-DNA adduct levels in different organs of mice exposed to AA-I or AA-II.

## Materials and Methods

### Caution

AAs are human carcinogens and should be handled with care.

### Chemicals and Materials

All reagents were of the highest purity available and were used without further purification. AA-I was purchased from Acros (Morris Plains, NJ). Benz[*cd*]indol-2(1*H*)-one, GSH (oxidized and reduced), [^13^C_2_,^15^N]-GSH, AA-II, 8-oxo-dG, alkaline phosphatase, DNase I, and nuclease P1 were obtained from Sigma (St. Louis, MO). Snake venom phosphodiesterase was obtained from US Biological (Swampscott, MA). Aristolactam I (AL-I), aristolactam II (AL-II), 7-(deoxyadenosin-*N*^6^-yl)-aristolactam I (ALI-dA; Scheme 1), 7-(deoxyadenosin-*N*^6^-yl)-aristolactam II (ALII-dA), [^15^N_5_]-labeled AA-DNA adduct internal standards (^15^N_5_-ALI-dA and ^15^N_5_-ALII-dA), and [^13^C_4_,^15^N_2_]-GSSG were from previous studies.^39−41^^15^N_5_-8-oxo-dG was purchased from Cambridge Isotope Laboratories, Inc. (Tewksbury, MA). Jejunum used in the noneverted gut sac experiment was freshly prepared from male mice, as stated in the previous study.^42−44^ LC–MS-grade acetonitrile was acquired from Tedia (Fairfield, OH). Deionized water was further purified with a laboratory water purification system (Cascada, PALL; Port Washington, NY) and was used in all of the experiments.

### Instrumentation

HPLC-FLD analysis of AL-I and AL-II was conducted on an UltiMate 3000 HPLC system (Thermo Fisher; Waltham, MA). LC–MS/MS analysis of GSH, oxidized glutathione (GSSG), AA-I, and AA-II was performed on a Waters Xevo TQD LC–MS/MS system (Milford, MA). Quantitative analysis of AA-DNA adducts and 8-oxo-dG was performed on a Waters Xevo TQ-XS LC–MS/MS system with a standard electrospray ionization source. A Luna C18 column (100 × 2 mm, 3 μm; Phenomenex; Torrance, CA) was used in all above analyses, with the respective mobile phase composition, LC gradient, and MS parameters listed in Table S1.

### Cell Culture

HEK293 and L02 cells (ATCC; Camden, NJ) were cultured in Dulbecco’s modified Eagle’s medium (Thermo Fisher; Waltham, MA) in a CO_2_ chamber, as described previously.^6,38,45^ After growing to ∼70% confluency (∼1.0 × 10^6^), different amounts of AA-I or AA-II (20, 30, 50, or 80 μM; separately and in combination; *n* = 3) were added to the medium.

### Absorptivity and Toxicity Tests

Forty-eight hours after the AA-I and/or AA-II addition, the cell culture medium was collected for AL-I/AL-II analysis. Cells were then detached by trypsin–EDTA, and the cell mortality rates were determined using the trypan-blue staining assay, after which cells were processed for AA-DNA adduct, 8-oxo-dG, GSH, and AA/AL analyses using our previously developed LC–MS/MS methods.^6,38,45−48^ Cells after exposure to 30 μM of AA-I and/or AA-II were also put to a fresh AA-free medium, and the cells were harvested at different times to study the cellular AA-DNA adduct levels. Similar experiments were also conducted on cells that were exposed to 30 μM of AA-I and/or AA-II for different durations (24, 48, and 72 h; *n* = 3) and in L02 cells (ATCC).

### Mice Experiment

Animal experiments were conducted following an approved protocol from the Animal Ethics Committee at HKUST (AEP number: A20002). Fifteen male C57BL/6 mice, aged 8–9 weeks, were obtained from the Laboratory Animal Facility, HKUST. After a week of acclimatization, the mice were randomly divided into three groups. One group received 10 mg/kg of AA-I, one group received 10 mg/kg of AA-II, both dissolved in 1% NaHCO_3_, and a control group received the dosing vehicle. After 24 h, the mice were sacrificed by decapitation, and their organs were collected for AA-DNA adduct analysis, as described previously.^3,39^

### Noneverted Gut Sac Experiment

Freshly prepared intestinal (the jejunum; 5 to 8 cm) segments were rinsed with cold Tyrode’s solution and filled with 150 μL of 1.0 μM AA-I (*n* = 5) or AA-II (*n* = 5) dissolved in oxygenated Tyrode’s solution. The ends were ligated to form a sac. Each sac was placed in a glass test tube with 8 mL of oxygenated Tyrode’s solution and maintained at 37 °C in a water bath (Figure S1). Samples (200 μL) of the solution outside the sacs were collected at 15, 30, 45, 60, 90, and 120 min. The samples were extracted three times with ethyl acetate, and the extracts were dried. The residues were then resuspended in 50 μL of 70% methanol with 60 nM of the internal standard benz[*cd*]indol-2(1*H*)-one and analyzed using LC–MS/MS for quantification of AAs.

### Sample Preparation and Analysis

#### AL-I and AL-II Analysis

0.8 mL of cell culture medium was collected and extracted thrice using 1 mL ethyl acetate. The organic extracts were combined and dried under nitrogen stream, before being redissolved in 0.1 mL methanol for AL-I and AL-II analysis using our previously developed HPLC-FLD method.^18,46^

#### AA and GSH Analyses

The AA exposed cells after being washed three times with PBS were lysed by ultrasonication in 0.2 mL PBS containing 1 nM butylated hydroxytoluene (BHT), 60 nM benz[*cd*]indol-2(1*H*)-one, and 10 μM [^13^C_2_,^15^N]-GSH and [^13^C_4_,^15^N_2_]-GSSG internal standards, centrifuged at 13,800 rcf at 4 °C, before the supernatant was collected for AA and GSH analysis, as described previously.^41,45−47^

#### AA-DNA Adduct and 8-Oxo-dG Analysis

In another experiment, the DNA of AA-exposed cells was isolated by an Omega Biotek DNA isolation kit (Norcross, GA) using the protocol suggested by the manufacturer, with modifications. The isolated DNA (∼15 μg dissolved in 100 μL water containing 1 nM BHT) was added with 15 μL of the internal standard solution mixture (containing 0.1 nM ^15^N_5_-ALI-dA, 0.1 nM ^15^N_5_-ALII-dA, and 1 nM ^15^N_5_-8-oxo-dG) before being digested with nuclease P1, DNase I, alkaline phosphatase, and snake venom phosphodiesterase, as described previously.^6,38,45,47,48^ The DNA hydrolysates after centrifugation at 13,800 rcf at 4 °C were analyzed using our previously developed LC–MS/MS coupled with an isotope dilution method.^6,38,45,47,48^

### Statistical Analysis

All data from cells and mice studies were represented as the mean ± SD for three and five independent experiments, respectively, and were compared with the respective group using Student’s *t*-test at 95% confidence interval. For Student’s *t*-test, the level of significance to *p* < 0.05 was set and the data were compared by using a two-sided *t*-test for unpaired samples.

## Results and Discussion

### Cytotoxicity

**Figure 1:**
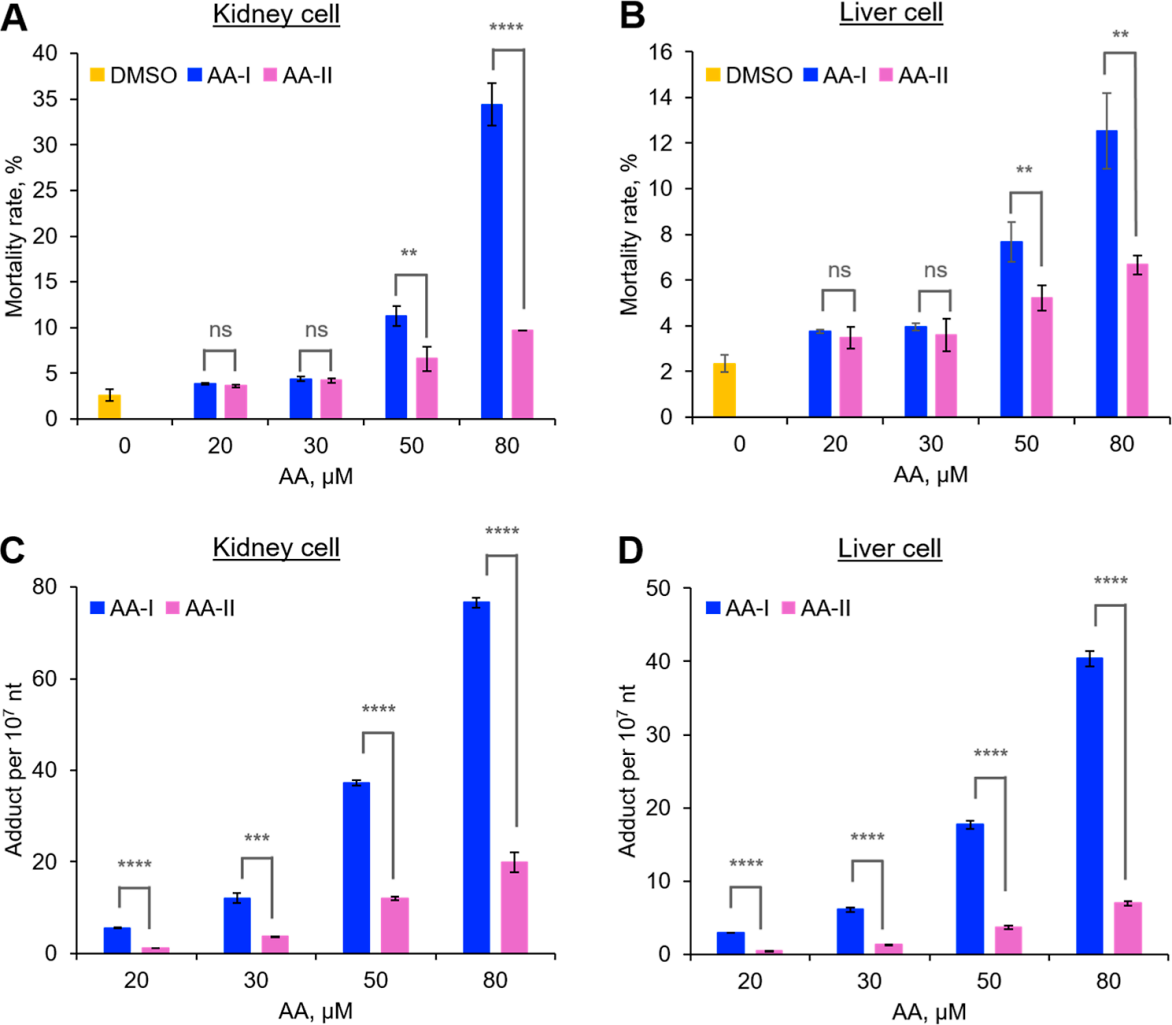
Cell mortality rates (A,B) and DNA adduct formation (C,D) in cultured kidney (A,C) and liver (B,D) cells that were exposed to different concentrations of AA-I or AA-II for 48 h. No AA-DNA adduct was detected in cells that were treated with the dosing vehicle (DMSO). Significant higher mortality rates and AA-DNA adduct formation were observed in AA-I than AA-II-exposed kidney and liver cells, with higher rates in kidney cells compared to liver cells. Student’s *t*-test where ns, *p* > 0.05; **, *p* < 0.01; ***, *p* < 0.001; ****, *p* < 0.0001. The data represent means ± SD for three independent experiments.

As shown in Figure 1A, exposure to AA-I and AA-II generally resulted in a concentration-dependent increase in the mortality rates in cultured human kidney cells. Specifically, there was no significant difference in cell mortality rates between cells exposed to 20 μM and 30 μM of either AA-I or AA-II. However, a significant increase in the cell mortality rate was observed in cells exposed to 50 μM and 80 μM of both AA-I and AA-II, with a more pronounced effect on cell survival observed in the AA-I-treated cells. Notably, the 48 h cell death rates were 34.4 ± 2.3% for AA-I and 9.7 ± 0.1% for AA-II at a concentration of 80 μM. It is important to mention that the concentration range chosen was based on preliminary study results, which indicated less than a 50% cell death rate at the highest applied concentration for AA-I. For ease comparison with AA-I, the same concentrations of AA-II were used in a parallel study.

Although observed to induce lower cell death rates than that in the kidney cells, a similar concentration dependence and higher mortality rates were also observed in AA-I than in AA-II-exposed liver cells (Figure 1B). These data indicated that AA-I exhibits higher cytotoxicity than AA-II in both human kidney and liver cells, with greater toxicity observed in kidney cells. The higher cytotoxic effect of AAs on kidney cells may help explain previous observations that the kidney is one of the primary target organs for AAs.^25,26,37,39^ Notably, this is the first report of the higher cytotoxicity of AA-I compared to that of AA-II.

### DNA Damage

We then investigated the mechanisms underlying the observed higher cytotoxicity of AA-I compared to that of AA-II, particularly regarding their differential toxicity to kidney and liver cells. Since DNA damage is believed to contribute to both tumor development and renal fibrotic processes associated with AAs,^36−38^ we initially quantified AA-DNA adducts, specifically ALI-dA and ALII-dA, in AA-exposed cells.

Similar to the cytotoxicity study, our analysis revealed a concentration-dependent formation of ALI-dA and ALII-dA in both AA-I- and AA-II-exposed kidney cells at all concentrations, with significantly higher adduct levels (∼3 times) observed for AA-I compared to those of AA-II (Figure 1C). Notably, no adducts were detected in control cells treated with the dosing vehicle only (DMSO), while 76.6 ± 1.1 ALI-dA per 10^7^ nt and 19.9 ± 2.1 ALII-dA per 10^7^ nt were detected in cells exposed to 80 μM AA-I and AA-II, respectively. Fitting the data by linear regression yielded lines with the following equations: ALI-dA: *y* = 1.2*x* – 21.7 (*r*^2^ = 0.99); ALII-dA: *y* = 0.32*x* – 5.2 (*r*^2^ = 0.99; Figure S2).

A time-dependent analysis of ALI-dA and ALII-dA adduct accumulation in AA-I- and AA-II-exposed cells provided further insights into the observed higher toxicity of AA-I. Specifically, as indicated by the exposure time-dependent accumulation of the AA-DNA adducts (*r*^2^ > 0.98; Figure S3) and steeper slope of the time-dependent curve for ALI-dA formation (0.22 vs 0.04, Figure S3A; and 0.22 vs 0.02, S3B), we detected faster adduct formation of ALI-dA in AA-I-exposed cells compared to ALII-dA in AA-II-exposed cells.

Analysis of DNA isolated from cultured liver cells revealed a similar phenomenon, with higher adduct levels for AA-I than for AA-II (Figure 1D) but at lower levels than those in kidney-isolated DNA. Fitting the data by linear regression yielded lines with the following equations: ALI-dA: *y* = 0.64*x* – 11.8 (*r*^2^ = 0.99; Figure S2); ALII-dA: *y* = 0.11*x* – 1.9 (*r*^2^ = 0.99). Given that the patterns of adduct formation and cell mortality rates are highly similar, it is likely that AA exposure-induced DNA adduct formation may have affected cell physiology, including the activities of DNA repair enzymes, ultimately leading to cell death.

**Figure 2:**
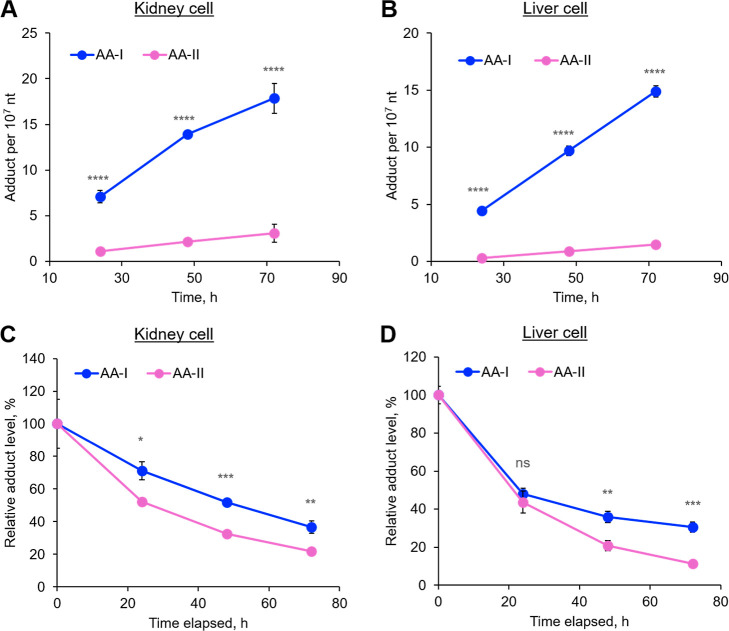
Exposure-time-dependent formation of the AA-DNA adduct (ALI-dA for AA-I; ALII-dA for AA-II) in cultured kidney (A) and liver (B) cells exposed to 30 μM of AA-I and AA-II for varying duration, along with the kinetics of AA-DNA adduct elimination from the AA-exposed kidney (C) and liver (D) cells. Faster AA-DNA adduct formation was observed for AA-I than AA-II in both kidney (A) and liver (B) cells. AA-DNA adducts formed by AA-I were eliminated at a slower rate than those formed by AA-II (C,D). Student’s *t*-test where ns, *p* > 0.05; *, *p* < 0.05; **, *p* < 0.01; ***, *p* < 0.001; ****, *p* < 0.0001. The data represent means ± SD for three independent experiments.

The hypothesis that AA-DNA adducts formed affect the activity of DNA repair enzymes is supported by the observation of slower elimination of ALI-dA compared to ALII-dA in AA-I- and AA-II-exposed cells, respectively (Figure 2C,D). ALI-dA appears to have a stronger impact on DNA repair enzyme activity than ALII-dA and/or is less efficiently repaired, resulting in ALI-dA being more persistent than ALII-dA. Consequently, AA-I exposure resulted in more ALI-dA accumulation and more cell death compared to AA-II (Figure 1).

### Oxidative Stress

In addition to AA-DNA adduct formation, emerging evidence suggests that AA exposure induced oxidative DNA damage and GSH depletion may play crucial roles in AAs’ toxicity.^47,48^ To better understand the observed differences in cytotoxicity between AA-I and AA-II, we quantified GSH levels and 8-oxo-dG, a major oxidative DNA damage product, in AA-I- or AA-II-exposed cells using LC–MS/MS coupled with stable isotope dilution methods.

**Figure 3:**
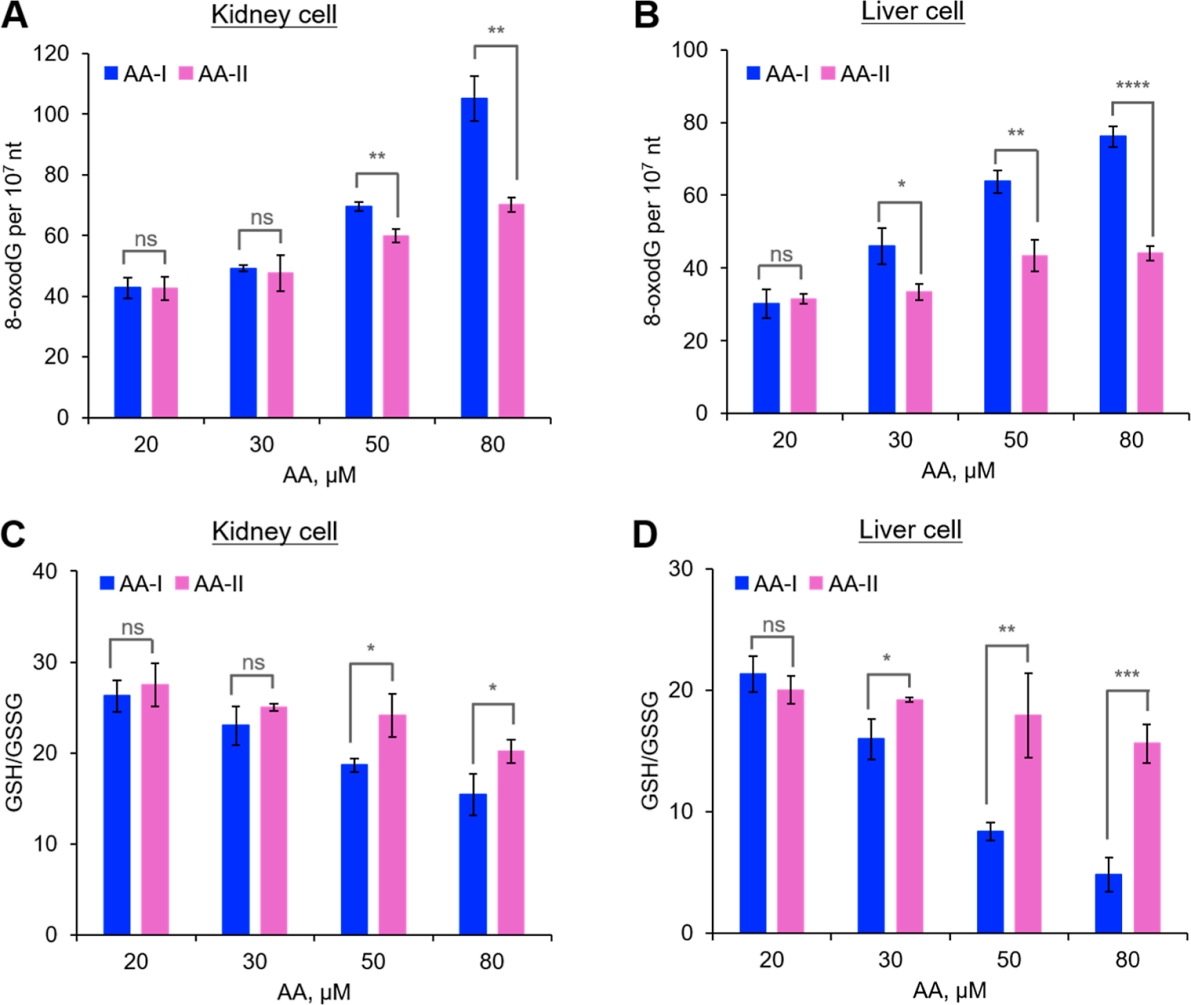
8-Oxo-dG (A,B) and GSH/GSSG ratio (C,D) in cultured kidney (A,C) and liver (B,D) cells exposed to different concentrations of AA-I or AA-II for 48 h. Higher 8-oxo-dG frequencies and GSH depletion were observed in cells exposed to AA-I compared to AA-II. Higher 8-oxo-dG frequencies and GSH depletion were also observed in kidney cells compared to liver cells. Student’s *t*-test where ns, *p* > 0.05; *, *p* < 0.05; **, *p* < 0.01; ***, *p* < 0.001; ****, *p* < 0.0001. The data represent means ± SD for three independent experiments.

Similar to the AA-DNA adduct analysis, the results revealed a concentration-dependent formation of 8-oxo-dG in the DNA isolated from exposed cells (*r*^2^ > 0.82; Figure S4), with higher concentrations observed in AA-I-exposed cells compared to AA-II-exposed cells (Figure 3A,B), and higher levels in kidney cells than in liver cells. Previous studies have shown that 8-oxo-dG lesions may lead to cell death,^48^ indicating that oxidative 8-oxo-dG lesions induced by AA exposure may partly account for the differing cytotoxicity of AA-I and AA-II.

A similar reduction in the GSH/GSSG ratio was observed in AA-I-exposed cells compared with AA-II-exposed cells (Figure 3C,D). GSH, one of the most abundant antioxidants, is oxidized to GSSG under oxidative stress conditions.^41,49^ The more pronounced decrease in GSH/GSSG ratios indicated that AA-I is a stronger inducer of oxidative stress than AA-II. Given that oxidative stress-induced DNA damage and GSH depletion are well studied for their potential roles in the development of human diseases, including cancer,^49^ these results suggest that the differing oxidative stress-inducing capabilities may have contributed to the observed differences in cytotoxicity between AA-I and AA-II.

### Absorptivity and Metabolic Activation of AA-I and AA-II

AAs, which have a hydrophobic domain and are anionic, are typical substrates for organic anion transporters 1 (OAT1) and 3 (OAT3).^50,51^ However, studies indicated that OAT1 and OAT3 transport AA-I and AA-II with similar efficiency.^51,52^ Thus, the difference in toxicity between AA-I and AA-II is likely due to factors beyond the affinity of these transporters. To further understand the mechanisms underlying the observed differences in cell viability, AA-DNA adduct formation, and oxidative stress between AA-I and AA-II, we investigated the absorption and metabolic activation of both compounds in exposed cells, as these are key factors affecting xenobiotic toxicity.

**Figure 4:**
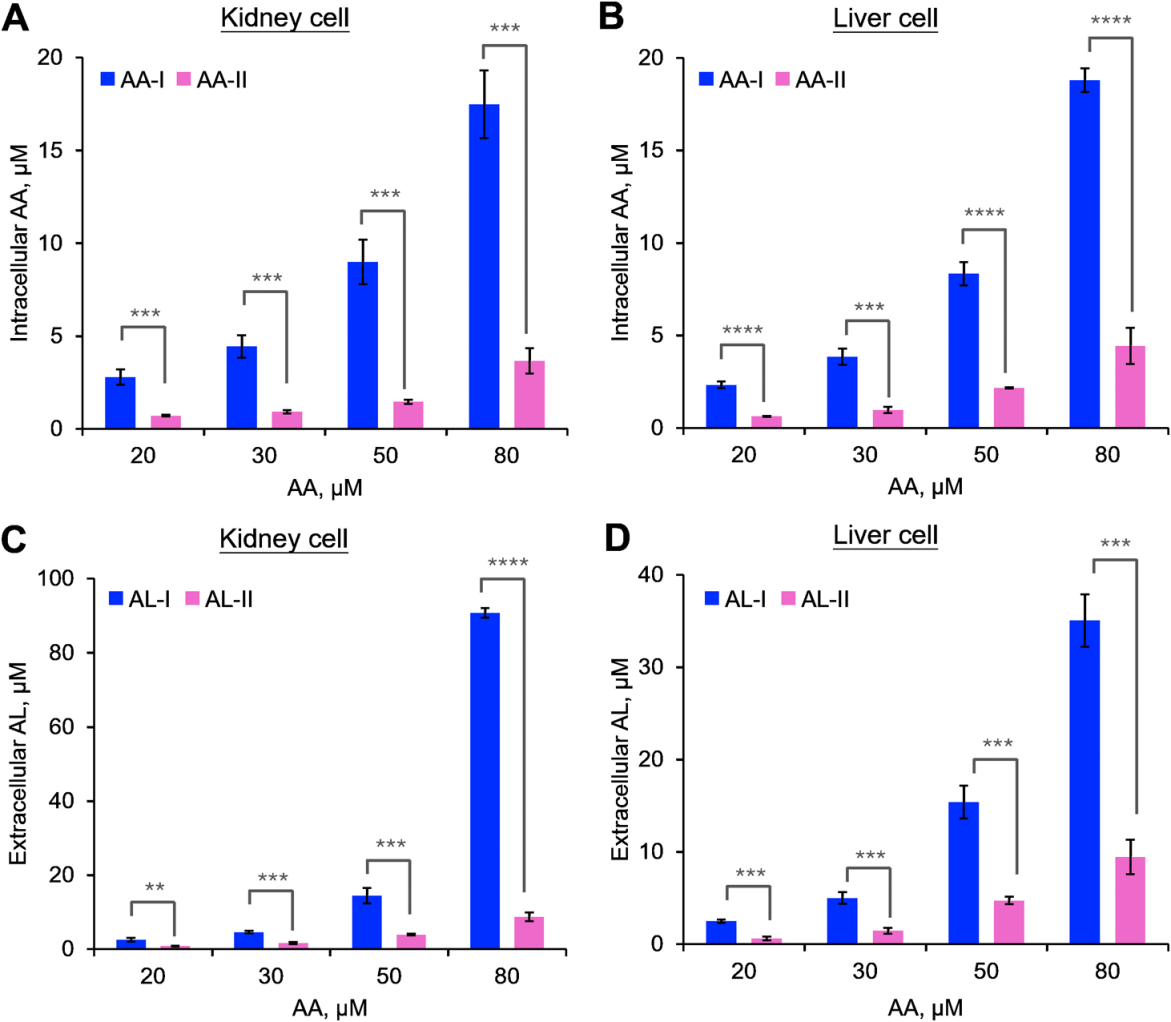
Concentrations of AA-I and AA-II in intracellular fluid of cultured kidney (A) and liver (B) cells exposed to different concentrations of AA-I or AA-II for 48 h, along with the concentrations of AL-I or AL-II in the cell culture medium (C,D). Higher cellular absorptivity of AA-I compared to AA-II was observed in the exposed cells. A higher reductive metabolism rate was observed for AA-I compared to AA-II. Student’s *t*-test where **, *p* < 0.01; ***, *p* < 0.001; ****, *p* < 0.0001. Fitting the data by linear regression yielded lines with the following equations. (A) AA-I: *y* = 0.25*x* – 2.7 (*r*^2^ = 0.99), AA-II: *y* = 0.050*x* – 0.54 (*r*^2^ = 0.94); (B) AA-I: *y* = 0.28*x* – 4.2 (*r*^2^ = 0.97), AA-II: *y* = 0.065*x* – 0.87 (*r*^2^ = 0.99); (C) AL-I: *y* = 1.5*x* – 38.7 (*r*^2^ = 0.87), AL-II: *y* = 0.13*x* – 2.2 (*r*^2^ = 0.99); and (D) AL-I: *y* = 0.56*x* – 10.5 (*r*^2^ = 0.99), AL-II: *y* = 0.15*x* – 2.7 (*r*^2^ = 0.99). The data represent means ± SD for three independent experiments.

As an indicator of absorptivity, we measured the concentrations of AA-I and AA-II in the exposed cells using LC–MS/MS. Analysis of the intracellular fluid from AA-exposed cells revealed a concentration-dependent accumulation of both AA-I and AA-II (*r*^2^ > 0.94), with significantly stronger absorptivity (∼3 times) for AA-I compared to that of AA-II at all tested concentrations (Figures 4 and S5). This finding aligns with the observed higher cytotoxicity (Figure 1), DNA adduct formation (Figure 1), and oxidative stress (Figure 3) associated with AA-I. Notably, previous studies have observed a dose-dependent relationship between cumulative AA exposure and the risk of developing end-stage renal disease in human.^11,22^ Therefore, in addition to previously reported results showing higher activity of NQO1 for the metabolic activation of AA-I compared to AA-II,^31^ these findings indicated for the first time that the stronger cellular absorption of AA-I may be responsible for its greater cytotoxicity.

The difference in absorption between AA-I and AA-II may be attributed to the stronger partitioning of AA-I in the hydrophobic phospholipid bilayer of cell membranes, as indicated by the 10-fold larger *n*-octanol–water partition coefficient of AA-I compared to AA-II reported in our previous study.^19^ As a result, intracellular AA-I concentrations are consistently higher than the intracellular AA-II concentrations. However, the intracellular concentrations of either AA-I or AA-II are of similar magnitude in both kidney and liver cells, suggesting the difference in toxicity in the two types of cells, as noted above, is not solely due to intracellular concentrations but rather must involve other factors, such as metabolizability.

To better understand the reasons for the different cytotoxicities of AA-I and AA-II in kidney and liver cells, we measured their major metabolites in the metabolic activation pathway. The key pathway involves nitroreduction (Scheme 1), which produces a reactive nitrenium ion that can form DNA adducts or aristolactams as end products.^11,23,24^ We specifically quantified AL-I and AL-II in the culture medium of cells exposed to AA-I and AA-II.

**Figure 5:**
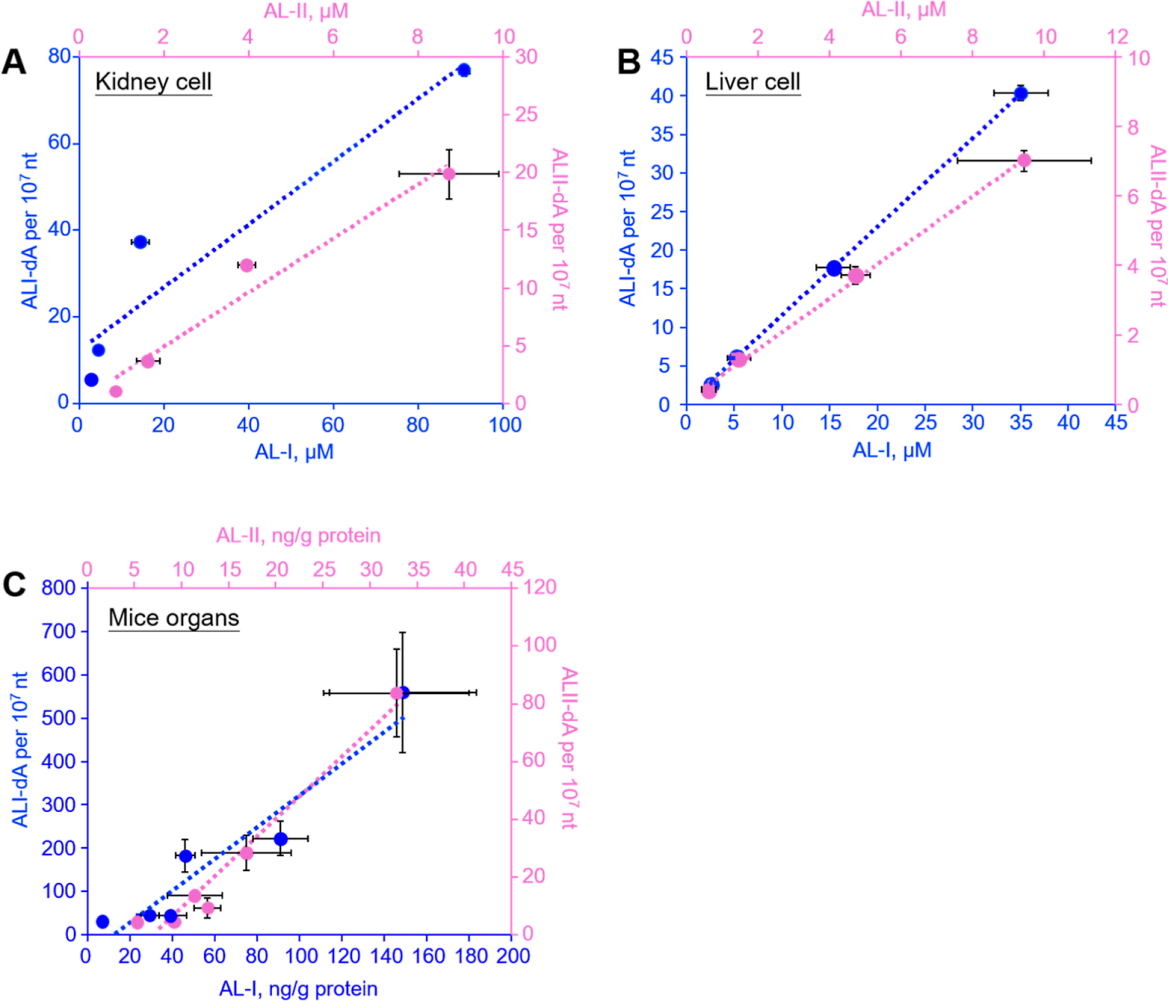
Correlation of the concentrations of DNA adducts with ALs in the culture medium of (A) kidney and (B) liver cells exposed to different concentrations of AAs and (C) in organs of mice that were treated with 10 mg/kg of AA-I or AA-II. Fitting the data by linear regression yielded lines with the following equations. (A) AA-I: *y* = 0.73*x* + 12.4 (*r*^2^ = 0.90), AA-II: *y* = 2.3*x* + 0.29 (*r*^2^ = 0.96); (B) AA-I: *y* = 1.1*x* + 0.22 (*r*^2^ = 0.99), AA-II: *y* = 0.73*x* + 0.14 (*r*^2^ = 0.99); and (C) AA-I: *y* = 3.8*x* + 46.7 (*r*^2^ = 0.90), AA-II: *y* = 2.9*x* + 14.4 (*r*^2^ = 0.95). The data represent means ± SD for three and five independent cell and mice experiments, respectively.

Our analysis revealed that AL-I levels were higher than AL-II in both kidney and liver cells (Figure 4C,D), which aligns well with the AA-DNA adduct levels (Figure 1C,D). Notably, both ALs, especially AL-I, were found in greater amounts in kidney cells compared to liver cells and a close correlation of AL levels with the AA-DNA adduct levels was observed (Figure 5; *r*^2^ > 0.90). This suggests that kidney cells have a slightly higher capacity for reducing AA-I, consistent with previous findings of greater NQO1 activity in the kidneys than in the liver.^53^

Although the liver is known for drug metabolism, it is not a primary target for AAs. The greater reductive metabolic activation of AA-I than AA-II in kidney cells may help explain the observed differences in cytotoxicity of AA-I and AA-II, supporting earlier findings that AAs are more likely to target the kidneys in tumor development in laboratory animals and human.^25,26,37,39^

### Effect of Coexposure to AA-I and AA-II on the Toxicity of AA-I and AA-II

As mentioned in the Introduction, AA-I and AA-II are the primary components of the AA family and are commonly found together in various *Aristolochia* and *Asarum* herbs.^14,21,22^ To simulate real-world exposure, we examined the impact of coexposure to AA-I and AA-II on their toxicity. We focused on measuring DNA adducts, which serve as indicators of nephrotoxicity and carcinogenicity, in kidney and liver cells that were exposed to both compounds.

**Figure 6:**
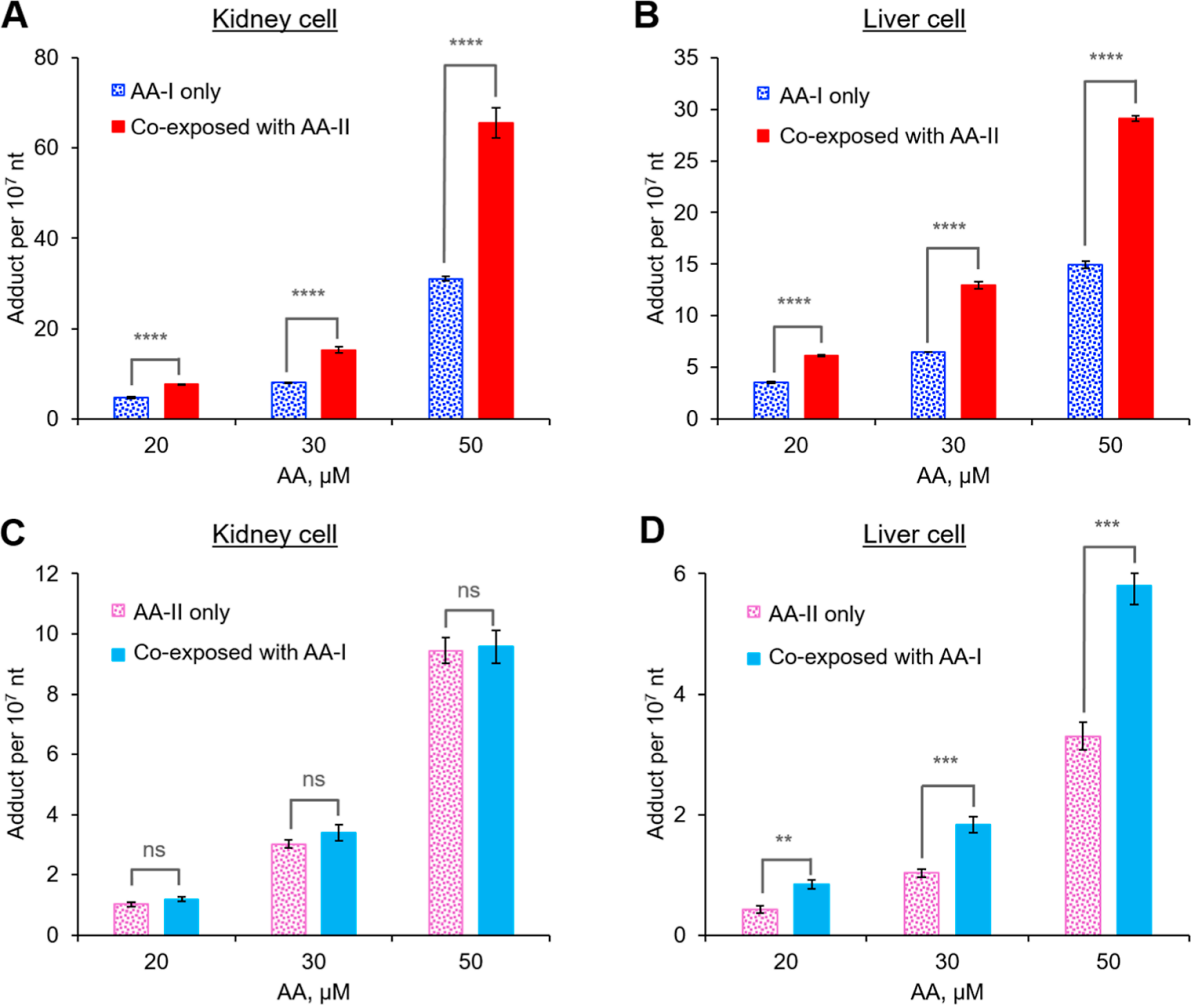
DNA adduct formation in cultured kidney (A,C) and liver (B,D) cells exposed to mixtures of AA-I (ALI-dA; A,B) and AA-II (ALII-dA; C,D) at various concentrations for 48 h. Significantly higher levels of AA-DNA adducts were observed in cells coexposed to the AA-I and AA-II mixture compared to those exposed to the same concentrations of AA-I or AA-II separately. Student’s *t*-test where ns, *p* > 0.05; **, *p* < 0.01; ***, *p* < 0.001; ****, *p* < 0.0001. Fitting the data by linear regression yielded lines with the following equations. (A) ALI-dA: *y* = 2.0*x* – 37.7 (*r*^2^ = 0.96) vs *y* = 0.92*x* – 16.0 (*r*^2^ = 0.95); (B) ALI-dA: *y* = 0.77*x* – 9.7 (*r*^2^ = 0.99) vs *y* = 0.39*x* – 4.5 (*r*^2^ = 0.99); (C) ALII-dA: *y* = 0.29*x* – 5.1 (*r*^2^ = 0.99) vs *y* = 0.28*x* – 4.7 (*r*^2^ = 0.99); and (D) ALII-dA: *y* = 0.17*x* – 2.9 (*r*^2^ = 0.98) vs *y* = 0.099*x* – 1.7 (*r*^2^ = 0.98). The data represent means ± SD from three independent experiments.

With the exception of ALII-dA (formed by AA-II) in kidney cells coexposed to both AA-I and AA-II, our results indicated that coexposure significantly increased the formation of ALI-dA and ALII-dA adducts in both kidney and liver cells (Figure 6). Specifically, while coexposure to AA-I had little effect on the formation of ALII-dA from AA-II in kidney cells, the presence of either AA-I or AA-II doubled the formation of their respective DNA adducts at all tested concentrations in both cell types.

These results are in good agreement with those observed in a previous study, which found that coexposure to AA-I and AA-II led to increased NQO1 activity in the kidneys and livers of mice.^54^ As DNA damage is believed to contribute to both nephrotoxicity and carcinogenicity associated with AAs,^36−38^ these findings suggest that the toxicity of these compounds increases when they are present together. This situation mirrors the real exposure risks faced by farmers in regions affected by BEN, who may consume homemade bread made from flour contaminated with a mixture of AAs.^1,16−19,55^

Subsequent analysis of the cellular AAs and ALs in the culture medium revealed that coexposure stimulated and increased both absorption of AAs and their reductive metabolic activation efficiency (Figures S6 and S7). This enhancement likely contributed to the observed increase in AA-DNA adduct formation in the AA-I/AA-II coexposed cells, which was also associated with a rise in cell death (Figure S8).

### Effects of AA-I or AA-II on Exposed Mice

**Figure 7:**
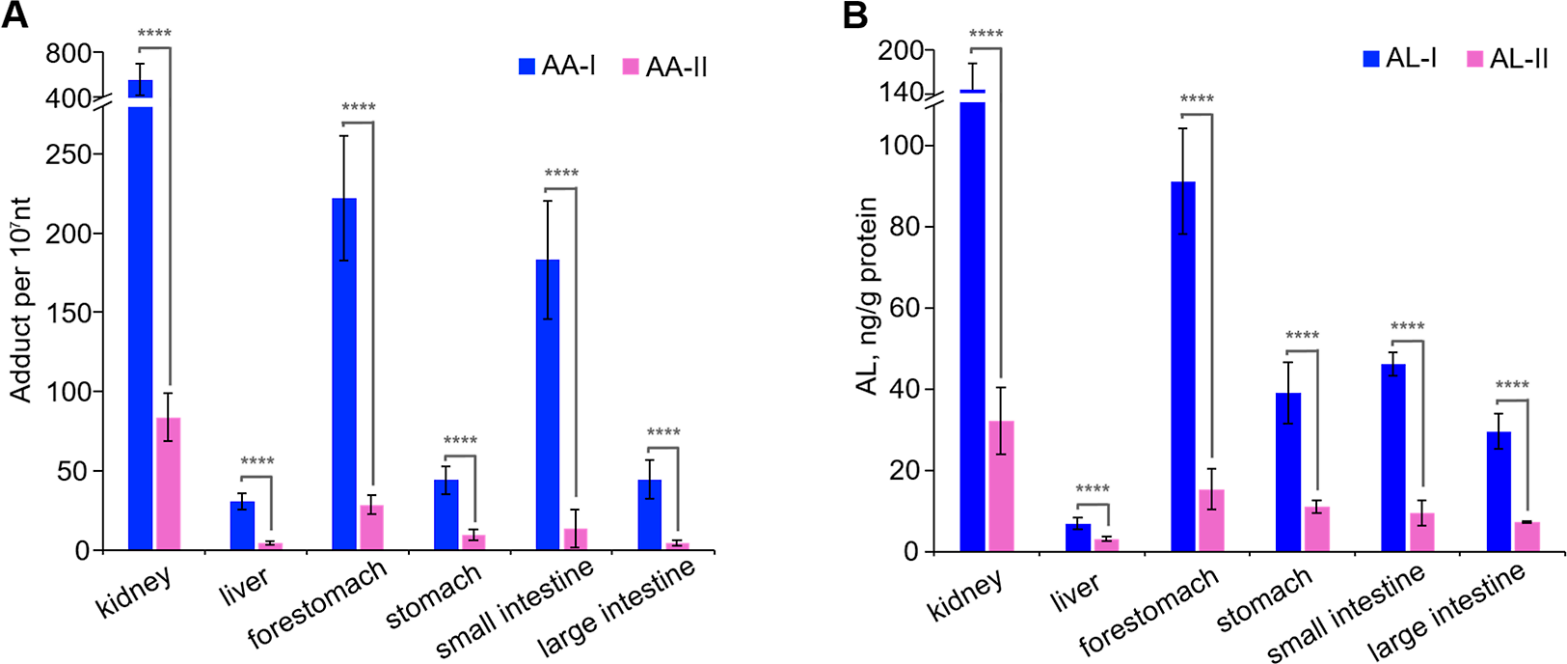
Concentrations of (A) ALI-dA and ALII-dA and (B) AL-I and AL-II in different organs of AA-I- or AA-II-exposed mice, respectively. Student’s *t*-test where ****, *p* < 0.0001. The data represent means ± SD for five independent experiments. No AA-DNA adducts or ALs were detected in internal organs of control mice that were dosed with the dosing vehicle.

After completing the cell studies, we aimed to quantify the concentrations of ALI-dA and ALII-dA in DNA isolated from various internal organs of mice that were given a single oral dose of 10 mg/kg of AA-I or AA-II. The analysis revealed that ALI-dA levels were significantly higher (approximately 10 times) than ALII-dA levels in the kidney, liver, forestomach, stomach, small intestine, and large intestine of mice exposed to AA-I compared to those exposed to AA-II (Figure 7). This indicated that AA-I has greater genotoxicity than AA-II in mice.

The highest levels of ALI-dA were found in DNA from the kidney (559.8 ± 139.2 adducts per 10^7^ nt), forestomach (221.9 ± 39.4 adducts per 10^7^ nt), and small intestine (183.0 ± 37.4 adducts per 10^7^ nt) of the AA-I-exposed mice. A correlative analysis of the adduct levels in the organs with that of the AL levels showed excellent correlation (*r*^2^ > 0.90; Figure 5C). These findings are consistent with previous observations that AAs target the kidney and forestomach for tumor development, and that AA-I is linked to the induction of carcinoma in the gastrointestinal tract.^26,39^

**Figure 8:**
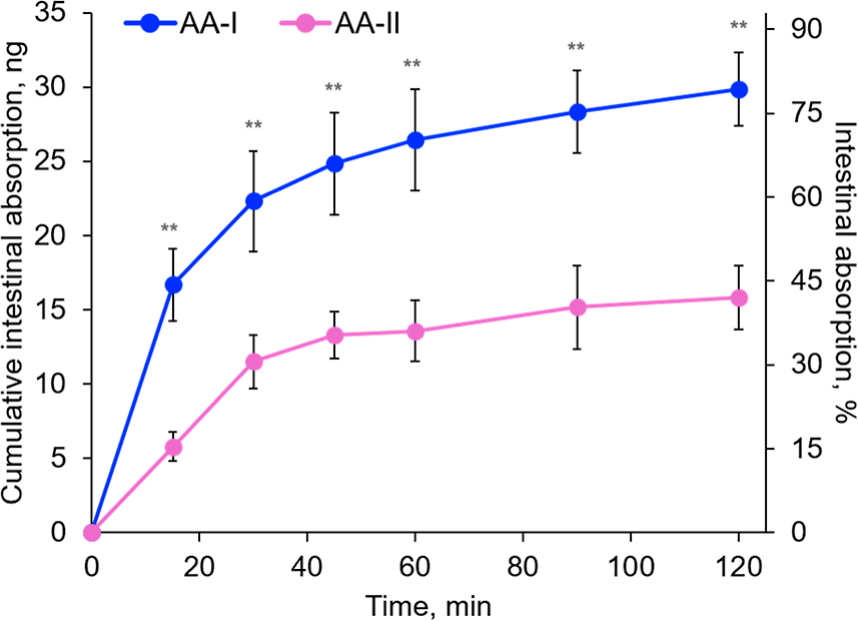
Time-course cumulative absorption of AA-I and AA-II in Tyrode’s solution outside the gut sacs, which contain 1 μM of AA-I and AA-II, respectively. Student’s *t*-test where **, *p* < 0.01. The data represent means ± SD for five independent experiments.

Because results from cell experiment indicated higher cell uptake of AA-I than AA-II (Figure 4A,B), we proceed to test the intestinal absorption of AA-I and AA-II using the noneverted gut sac method using freshly prepared mouse jejunum, as described in the Material and Methods section. Similar to that observed in the cell studies, the study showed significantly faster and higher absorption of AA-I than AA-II, with approximately 80% and 40% being Tyrode’s solution outside the gut sacs in the 2 h experiment, respectively (Figure 8). These results reinforced our conclusion that the differences in absorptivity may be a previously unaware but important factor in the toxicity of AAs.

Using chemical methods, we demonstrated that AA-I is significantly more cytotoxic than AA-II, with a cell mortality rate of 34.4 ± 2.3% for AA-I compared to 9.7 ± 0.1% for AA-II at 80 μM (*p* < 0.0001). The data suggest that this increased cytotoxicity is partly due to AA-I’s higher rate of DNA adduct formation (approximately three times greater for AA-I than for AA-II; *p* < 0.001) and its stronger induction of oxidative stress. Analysis of the intracellular fluid from cells exposed to AA-I or AA-II, along with intestinal absorptivity experiments, revealed significantly higher absorption of AA-I, indicating approximately three times greater cellular uptake compared to AA-II (*p* < 0.001). Previous studies have highlighted the higher activity of metabolic activation enzymes, such as NQO1, as a key reason for AA-I’s greater nephrotoxicity and carcinogenicity.^31^ Our study contributes to this understanding by presenting evidence that the significantly higher absorption of AA-I is a previously unrecognized factor in its increased toxicity. Furthermore, we found significantly higher levels of AA-DNA adducts in DNA isolated from mice exposed to AA-I. We believe these results will enhance our understanding of the pronounced toxic effects associated with AA-I and that future risk assessments of AA exposure may specifically target AA-I.

## Supporting Information Available

Mobile-phase composition and LC gradient for HPLC-FLD of AL-I and AL-II; optimized ESI-MS parameters for LC–MS/MS analysis of AA-I, AA-II, GSH, GSSG, AA-DNA adducts, and 8-oxo-dG; schematic illustration of the noneverted gut sac study setup used in this study; cell mortality rates, absorption, and metabolism of cells that were exposed to a mixture of AA-I and AA-II; linear regression analysis of AA absorption, ALs, DNA adduct, and 8-oxo-dG; and formation in cells exposed to different concentration of AAs (PDF)

## Abbreviations

AAs: aristolochic acids
AA-I: aristolochic acid I
AA-II: aristolochic acid II
ALI-dA: 7-(deoxyadenosin-*N*^6^-yl)-aristolactam I
ALII-dA: 7-(deoxyadenosin-*N*^6^-yl)-aristolactam II
BEN: Balkan endemic nephropathy
LC–MS/MS: liquid chromatography–tandem mass spectrometry.

## References

[ref1] Pavlovic N. M. Balkan endemic nephropathy—current status and future perspectives. Clin. Kidney J. 2013, 6, 257–265. 10.1093/ckj/sft049. 26064484 PMC4400492

[ref2] Batuman V. Fifty years of Balkan endemic nephropathy: Daunting questions, elusive answers. Kidney Int. 2006, 69, 644–646. 10.1038/sj.ki.5000231. 16467889

[ref3] Ham Y.-H.; Chin M.-L.; Pan G.; Wang S.; Pavlović N. M.; Chan W. Positive feedback mechanism in aristolochic acid I exposure-induced anemia and DNA adduct formation: Implications for Balkan endemic nephropathy. J. Agric. Food Chem. 2024, 72, 18155–18161. 10.1021/acs.jafc.4c03508. 39088813

[ref4] Dimitrov P.; Tsolova S.; Georgieva R.; Bozhilova D.; Simeonov V.; Bonev A.; Karmaus W. Clinical markers in adult offspring of families with and without Balkan endemic nephropathy. Kidney Int. 2006, 69, 723–729. 10.1038/sj.ki.5000120. 16407881

[ref5] Grollman A. P.; Shibutani S.; Moriya M.; Miller F.; Wu L.; Moll U.; Suzuki N.; Fernandes A.; Rosenquist T.; Medverec Z.; Jakovina K.; Brdar B.; Slade N.; Turesky R. J.; Goodenough A. K.; Rieger R.; Vukelić M.; Jelaković B. Aristolochic acid and the etiology of endemic (Balkan) nephropathy. Proc. Natl. Acad. Sci. U.S.A. 2007, 104, 12129–12134. 10.1073/pnas.0701248104. 17620607 PMC1913550

[ref6] Guo W.; Kwok H.-C.; Griffith S. M.; Nagl S.; Milovanović D.; Pavlović M.; Pavlović N. M.; Yu J. Z.; Dedon P. C.; Chan W. Combustion-derived pollutants linked with kidney disease in low-lying flood-affected areas in the Balkans. Environ. Sci. Technol. 2024, 58, 11301–11308. 10.1021/acs.est.4c02848. 38900968

[ref7] Stefanović V.; Polenaković M. Fifty years of research in Balkan endemic nephropathy: Where are we now?. Nephron Clin. Pract 2009, 112, c51–c56. 10.1159/000213081. 19390202

[ref8] Feder G. L.; Radovanović Z.; Finkelman R. B. Relationship between weathered coal deposits and the etiology of Balkan endemic nephropathy. Kidney Int. 1991, 40, 4. 1762344

[ref9] Orem W. H.; Feder G. L.; Finkelman R. B. A possible link between Balkan endemic nephropathy and the leaching of toxic organic compounds from Pliocene lignite by groundwater: Preliminary investigation. Int. J. Coal Geol. 1999, 40, 237–252. 10.1016/S0166-5162(98)00071-8.

[ref10] Tatu C. A.; Orem W. H.; Finkelman R. B.; Feder G. L. The etiology of Balkan endemic nephropathy: Still more questions than answers. Environ. Health Perspect. 1998, 106, 689–700. 10.1289/ehp.106-1533478. PMC1533478 9799184

[ref11] Stiborová M.; Arlt V. M.; Schmeiser H. H. Balkan endemic nephropathy: An update on its aetiology. Arch. Toxicol. 2016, 90, 2595–2615. 10.1007/s00204-016-1819-3. 27538407 PMC5065591

[ref12] Schiller A.; Gusbeth-Tatomir P.; Paylovic N.; Ferluga D.; Spasovski G.; Covic A. Balkan endemic nephropathy: A still unsolved puzzle. J. Nephrol. 2008, 21, 673. 18949721

[ref13] Jelaković B.; Karanović S.; Vuković-Lela I.; Miller F.; Edwards K. L.; Nikolić J.; Tomić K.; Slade N.; Brdar B.; Turesky R. J.; Stipančić Z. ˇ.; Dittrich D.; Grollman A. P.; Dickman K. G. Aristolactam-DNA adducts are a biomarker of environmental exposure to aristolochic acid. Kidney Int. 2012, 81, 559–567. 10.1038/ki.2011.371. 22071594 PMC3560912

[ref14] Debelle F. D.; Vanherweghem J. L.; Nortier J. L. Aristolochic acid nephropathy: A worldwide problem. Kidney Int. 2008, 74, 158–169. 10.1038/ki.2008.129. 18418355

[ref15] Ivić M. Etiology of endemic nephropathy. Lijec. Vjesn. 1969, 91, 1273–1281. 5200813

[ref16] Chan C.-K.; Liu Y.; Pavlović N. M.; Chan W. Etiology of Balkan endemic nephropathy: An update on aristolochic acids exposure mechanisms. Chem. Res. Toxicol. 2018, 31, 1109–1110. 10.1021/acs.chemrestox.8b00291. 30346143

[ref17] Li W.; Chan C.-K.; Liu Y.; Yao J.; Mitić B.; Kostić E. N.; Milosavljević B.; Davinić I.; Orem W. H.; Tatu C. A.; Dedon P. C.; Pavlović N. M.; Chan W. Aristolochic acids as persistent soil pollutants: Determination of risk for human exposure and nephropathy from plant uptake. J. Agric. Food Chem. 2018, 66, 11468–11476. 10.1021/acs.jafc.8b04770. 30286603 PMC6413692

[ref18] Chan W.; Pavlović N. M.; Li W.; Chan C.-K.; Liu J.; Deng K.; Wang Y.; Milosavljević B.; Kostić E. N. Quantitation of aristolochic acids in corn, wheat grain, and soil samples collected in Serbia: Identifying a novel exposure pathway in the etiology of Balkan endemic nephropathy. J. Agric. Food Chem. 2016, 64, 5928–5934. 10.1021/acs.jafc.6b02203. 27362729

[ref19] Li W.; Hu Q.; Chan W. Uptake and accumulation of nephrotoxic and carcinogenic aristolochic acids in food crops grown in *Aristolochia clematitis*-contaminated soil and water. J. Agric. Food Chem. 2016, 64, 107–112. 10.1021/acs.jafc.5b05089. 26654710

[ref20] Kwok H.-C.; Chan W. Aristolochic acid exposure via dermal contact or inhalation of herbal powders: Evidence of occupational exposure in herbalists with urothelial cancer. Chem. Res. Toxicol. 2024, 37, 873–877. 10.1021/acs.chemrestox.4c00157. 38780306

[ref21] Chan W.; Hui K. M.; Poon W. T.; Lee K. C.; Cai Z. Differentiation of herbs linked to “Chinese herb nephropathy” from the liquid chromatographic determination of aristolochic acids. Anal. Chim. Acta 2006, 576, 112–116. 10.1016/j.aca.2006.03.008. 17723621

[ref22] Jadot I.; Declèves A. E.; Nortier J.; Caron N. An integrated view of aristolochic acid nephropathy: Update of the literature. 2017. Int. J. Mol. Sci. 2017, 18, 297 10.3390/ijms18020297. 28146082 PMC5343833

[ref23] Chan W.; Cui L.; Xu G.; Cai Z. Study of the phase I and phase II metabolism of nephrotoxin aristolochic acid by liquid chromatography/tandem mass spectrometry. Rapid Commun. Mass Spectrom. 2006, 20, 1755–1760. 10.1002/rcm.2513. 16676316

[ref24] Krumbiegel G.; Hallensleben J.; Mennicke W. H.; Rittmann N.; Roth H. J. Studies on the metabolism of aristolochic acids I and II. Xenobiotica 1987, 17, 981–991. 10.3109/00498258709044197. 3673113

[ref25] Shibutani S.; Dong H.; Suzuki N.; Ueda S.; Miller F.; Grollman A. P. Selective toxicity of aristolochic acids I and II. Drug Metab. Dispos. 2007, 35, 1217–1222. 10.1124/dmd.107.014688. 17392392

[ref26] Pfau W.; Schmeiser H. H.; Wiessler M. 32P-postlabelling analysis of the DNA adducts formed by aristolochic acid I and II. Carcinogenesis 1990, 11, 1627–1633. 10.1093/carcin/11.9.1627. 2401053

[ref27] Das S.; Thakur S.; Korenjak M.; Sidorenko V. S.; Chung F. F. L.; Zavadil J. Aristolochic acid-associated cancers: A public health risk in need of global action. Nat. Rev. Cancer 2022, 22, 576–591. 10.1038/s41568-022-00494-x. 35854147

[ref28] Kucab J. E.; Zou X.; Morganella S.; Joel M.; Nanda A. S.; Nagy E.; Gomez C.; Degasperi A.; Harris R.; Jackson S. P.; Arlt V. M.; Phillips D. H.; Nik-Zainal S. A compendium of mutational signatures of environmental agents. Cell 2019, 177, 821–836. 10.1016/j.cell.2019.03.001. 30982602 PMC6506336

[ref29] Chiang S. Y.; Wey M. T.; Luo Y. S.; Shih W. C.; Chimeddulam D.; Hsu P. C.; Huang H. F.; Tsai T. H.; Wu K. Y. Simultaneous toxicokinetic studies of aristolochic acid I and II and aristolactam I and II using a newly-developed microdialysis liquid chromatography-tandem mass spectrometry. Food Chem. Toxicol. 2023, 177, 113856 10.1016/j.fct.2023.113856. 37257633

[ref30] Schmeiser H. H.; Schoepe K. B.; Wiessler M. DNA adduct formation of aristolochic acid I and II in vitro and in vivo. Carcinogenesis 1988, 9, 297–303. 10.1093/carcin/9.2.297. 3338114

[ref31] Martinek V.; Kubickova B.; Arlt V. M.; Frei E.; Schmeiser H. H.; Hudecek J.; Stiborova M. Comparison of activation of aristolochic acid I and II with NADPH: quinone oxidoreductase, sulphotransferases and N-acetyltranferases. Neuroendocrinol. Lett. 2011, 32, 57–70. 22167209

[ref32] Stiborová M.; Frei E.; Breuer A.; Wiessler M.; Schmeiser H. H. Evidence for reductive activation of carcinogenic aristolochic acids by prostaglandin H synthase—32P-postlabeling analysis of DNA adduct formation. Mutat. Res., Genet. Toxicol. Environ. Mutagen. 2001, 493, 149–160. 10.1016/S1383-5718(01)00171-1. 11516724

[ref33] Stiborová M.; Frei E.; Wiessler M.; Schmeiser H. H. Human enzymes involved in the metabolic activation of carcinogenic aristolochic acids: evidence for reductive activation by cytochromes P450 1A1 and 1A2. Chem. Res. Toxicol. 2001, 14, 1128–1137. 10.1021/tx010059z. 11511187

[ref34] Martínek V.; Bárta F.; Hodek P.; Frei E.; Schmeiser H. H.; Arlt V. M.; Stiborová M. Comparison of the oxidation of carcinogenic aristolochic acid I and II by microsomal cytochromes P450 in vitro: experimental and theoretical approaches. Monatshefte für Chemie-Chemical Monthly 2017, 148, 1971–1981. 10.1007/s00706-017-2014-9. PMC5653735 29104318

[ref35] Dedíková A.; Bárta F.; Martínek V.; Kotalík K.; Dušková S.; Mráz J.; Arlt V. M.; Stiborová M.; Hodek P. In vivo metabolism of aristolochic acid I and II in rats is influenced by their coexposure. Chem. Res. Toxicol. 2020, 33, 2804–2818. 10.1021/acs.chemrestox.0c00198. 32894017

[ref36] Cosyns J. P. Aristolochic acid and ‘Chinese Herbs Nephropathy’ a review of the evidence to date. Drug Saf. 2003, 26, 33–48. 10.2165/00002018-200326010-00004. 12495362

[ref37] Arlt V. M.; Stiborova M.; Schmeiser H. H. Aristolochic acid as a probable human cancer hazard in herbal remedies: a review. Mutagenesis 2002, 17, 265–277. 10.1093/mutage/17.4.265. 12110620

[ref38] Zhang J.; Chan K. K. J.; Chan W. Synergistic interaction of polycyclic aromatic hydrocarbons, phthalate esters, or phenol on DNA adduct formation by aristolochic acid I: Insights into the etiology of Balkan endemic nephropathy. Chem. Res. Toxicol. 2022, 35, 849–857. 10.1021/acs.chemrestox.2c00026. 35471859

[ref39] Liu Y.; Chan C.-K.; Jin L.; Wong S. K.; Chan W. Quantitation of DNA adducts in target and nontarget organs of aristolochic acid I-exposed rats: Correlating DNA adduct levels with organotropic activities. Chem. Res. Toxicol. 2019, 32, 397–399. 10.1021/acs.chemrestox.8b00359. 30604963

[ref40] Au C.-K.; Chan C.-K.; Tung K.-K.; Zhang J.; Chan W. Quantitation of DNA adducts of aristolochic acids in repair-deficient cells: A mechanistic study of the DNA repair mechanism. Chem. Res. Toxicol. 2020, 33, 1323–1327. 10.1021/acs.chemrestox.0c00004. 32115938

[ref41] Ham Y.-H.; Jason Chan K. K.; Chan W. Thioproline serves as an efficient antioxidant protecting human cells from oxidative stress and improves cell viability. Chem. Res. Toxicol. 2020, 33, 1815–1821. 10.1021/acs.chemrestox.0c00055. 32299210

[ref42] Zhang J.; Li W.; Liu Y.; He Y.; Cheng Z.; Li X.; Chen Y.; Zhang A.; Peng Y.; Zheng J. Arsenite-induced drug–drug interactions in rats. Drug Metab. Dispos. 2024, 52, 911–918. 10.1124/dmd.124.001772. 38849209

[ref43] Chen P.; Zhao M.; Chen Q.; Fan L.; Gao F.; Zhao L. Absorption characteristics of chitobiose and chitopentaose in the human intestinal cell line Caco-2 and everted gut sacs. J. Agric. Food Chem. 2019, 67, 4513–4523. 10.1021/acs.jafc.9b01355. 30929431

[ref44] Liu W.; Pan H.; Zhang C.; Zhao L.; Zhao R.; Zhu Y.; Pan W. Developments in methods for measuring the intestinal absorption of nanoparticle-bound drugs. Int. J. Mol. Sci. 2016, 17, 1171 10.3390/ijms17071171. 27455239 PMC4964542

[ref45] Zhang J.; Chan C.-K.; Pavlovic N. M.; Chan W. Effects of diet on aristolochic acid-DNA adduct formation: Implications for Balkan endemic nephropathy etiology. Chem. Res. Toxicol. 2023, 36, 438–445. 10.1021/acs.chemrestox.2c00370. 36881864

[ref46] Kwok H.-C.; Chan W. Wheatgrass (*Triticum aestivum*) as an efficient phytoremediation plant for aristolochic acid-contaminated water and arable soil. ACS Agric. Sci. Technol. 2022, 2, 639–645. 10.1021/acsagscitech.2c00057.

[ref47] Au C.-K.; Ham Y.-H.; Chan W. Bioaccumulation and DNA adduct formation of aristolactam I: Unmasking a toxicological mechanism in the pathophysiology of aristolochic acid nephropathy. Chem. Res. Toxicol. 2023, 36, 322–329. 10.1021/acs.chemrestox.2c00415. 36757010

[ref48] Chan W.; Ham Y.-H. Probing the hidden role of mitochondrial DNA damage and dysfunction in the etiology of aristolochic acid nephropathy. Chem. Res. Toxicol. 2021, 34, 1903–1909. 10.1021/acs.chemrestox.1c00175. 34255491

[ref49] Roy N.; Paira P. Glutathione depletion and stalwart anticancer activity of metallotherapeutics inducing programmed cell death: Opening a new window for cancer therapy. ACS Omega 2024, 9, 20670–20701. 10.1021/acsomega.3c08890. 38764686 PMC11097382

[ref50] Baudoux T. E.; Pozdzik A. A.; Arlt V. M.; De Prez E. G.; Antoine M. H.; Quellard N.; Goujon J. M.; Nortier J. L. Probenecid prevents acute tubular necrosis in a mouse model of aristolochic acid nephropathy. Kidney Int. 2012, 82, 1105–1113. 10.1038/ki.2012.264. 22854641

[ref51] Babu E.; Takeda M.; Nishida R.; Noshiro-Kofuji R.; Yoshida M.; Ueda S.; Fukutomi T.; Anzai N.; Endou H. Interactions of human organic anion transporters with aristolochic acids. J. Pharmacol. Sci. 2010, 113, 192–196. 10.1254/jphs.09339SC. 20508390

[ref52] Dickman K. G.; Sweet D. H.; Bonala R.; Ray T.; Wu A. Physiological and molecular characterization of aristolochic acid transport by the kidney. J. Pharmacol. Exp. Ther. 2011, 338, 588–597. 10.1124/jpet.111.180984. 21546538 PMC3141898

[ref53] Gaikwad A.; Long D. J.; Stringer J. L.; Jaiswal A. K. In vivo role of NAD (P) H: quinone oxidoreductase 1 (NQO1) in the regulation of intracellular redox state and accumulation of abdominal adipose tissue. J. Biol. Chem. 2001, 276, 22559–22564. 10.1074/jbc.M101053200. 11309386

[ref54] Bárta F.; Dedíková A.; Bebová M.; Dušková S. ˇ.; Mráz J.; Schmeiser H. H.; Arlt V. M.; Hodek P.; Stiborová M. Co-exposure to aristolochic acids I and II increases DNA adduct formation responsible for aristolochic acid I-mediated carcinogenicity in rats. Int. J. Mol. Sci. 2021, 22, 10479 10.3390/ijms221910479. 34638820 PMC8509051

[ref55] Jelaković B.; Dika Z. ˇ.; Arlt V. M.; Stiborova M.; Pavlović N. M.; Nikolić J.; Colet J.; Vanherweghem J.; Nortier J. L. Balkan endemic nephropathy and the causative role of aristolochic acid. Semin. Nephrol. 2019, 39, 284–296. 10.1016/j.semnephrol.2019.02.007. 31054628

