# Supplementary material for: Absorptivity Is an Important Determinant in the Toxicity Difference between Aristolochic Acid I and Aristolochic Acid II
Source: J Agric Food Chem. 2025 Jan 14;73(4):2551–61. doi: 10.1021/acs.jafc.4c10765 (PMC11800392; doi:10.1021/acs.jafc.4c10765)
Supplement: Supplementary file 1 — jf4c10765_si_001.pdf [file jf4c10765_si_001.pdf]

Supporting Information

Absorptivity is an Important Determinant in the Toxicity Difference between Aristolochic Acid I and Aristolochic Acid II

Hong-Ching Kwok[†], *Hei-Tak Tse*[†], *Ka-Ki Ng*[†], *Shuangshuang Wang*[†], *Chun-Kit Au*[†], *Zongwei Cai*[‡,#], *Wan Chan*[†,*]

[†] Department of Chemistry, The Hong Kong University of Science and Technology, Clear Water Bay, Kowloon, Hong Kong

[‡] Eastern Institute of Technology Ningbo, Zhejiang, China; Postal code 315200

[#] Department of Chemistry and State Key Laboratory of Environmental and Biological Analysis, Hong Kong Baptist University, Kowloon Tong, Kowloon, Hong Kong SAR, China

* Corresponding author

Email: chanwan@ust.hk; Phone: +852 2358-7370; Fax: +852 2358-1594.

TABLE OF CONTENTS

Table S1. LC gradient, MS source parameters, and MS compound parameters for LC-MS/MS analysis of (A) AA-DNA adducts and 8-oxo-dG, (B) AA-I and AA-II, (C) GSH and GSSG, and (D) HPLC-FLD analysis of AL-I and AL-II. (*Page S3 – S6*)

Figure S1. Schematic illustration of non-everted gut sac study set-up used in this study. (*Page S7*)

Figure S2. DNA adduct formation in cultured kidney (A) and liver (B) cells that were exposed to different concentrations of AA-I or AA-II for 48 h. (*Page S8*)

Figure S3. Exposure-time dependent formation of AA-DNA adduct in cultured kidney (A) and liver (B) cells exposed to of 30 μM of AA-I and AA-II for varying duration. (*Page S9*)

Figure S4. Level of 8-oxo-dG in cultured kidney (A) and liver (B) cells exposed to different concentrations of AA-I or AA-II for 48 h. (*Page S10*)

Figure S5. Concentrations of AA-I and AA-II in intracellular fluid of cultured kidney (A) and liver (B) cells exposed to different concentrations of AA-I or AA-II for 48 h. (*Page S11*)

Figure S6. Concentrations of AA-I (A and B) and AA-II (C and D) in intracellular fluid of cultured kidney (A and C) and liver (B and D) cells co-exposed to different concentrations of AA-I and AA-II for 48 h. (*Page S12*)

Figure S7. Concentrations of AL-I (A and B) and AL-II (C and D) in cell culture medium of cultured kidney (A and C) and liver (B and D) cells co-exposed to different concentrations of AA-I and AA-II for 48 h. (*Page S13*)

Figure S8. Cell mortality rates in cultured kidney (A) and liver (B) cells that were co-exposed to different concentrations of AA-I and AA-II for 48 h. (*Page S14*)

Table S1. LC gradient, MS source parameters, and MS compound parameters for LC-MS/MS analysis of (A) AA-DNA adducts and 8-oxo-dG, (B) AA-I and AA-II, (C) GSH and GSSG, and (D) HPLC-FLD analysis of AL-I and AL-II.

(A) AA-DNA adduct and 8-oxo-dG analysis

LC gradient

Time, min	Flow rate, mL/min	%A	%B
0.00	0.35	98.0	2.0
2.00	0.35	98.0	2.0
3.00	0.35	70.0	30.0
6.00	0.35	30.0	70.0
7.00	0.35	0.0	100.0
11.00	0.35	0.0	100.0
11.10	0.35	98.0	2.0
13.50	0.35	98.0	2.0

A: 0.1% acetic acid in water; B: acetonitrile

MS source parameter

Capillary Voltage, kV	2
Source Temperature, ^{o}C	150
Desolvation Temperature, ^{o}C	500
Cone gas flow, L/h	300
Desolvation gas flow, L/h	1000

MS compound parameter

	**Parent ion, *m/z***	**Daughter ion, *m/z***	**Cone voltage, V**	**Collision energy, eV**
ALI-dA	543	427	40	20
	543	292	40	40
ALII-dA	513	397	40	22
	513	380	40	42
8-oxo-dG	284	168	20	13
	284	117	20	13
$^{15}N_5$-ALI-dA	548	432	40	20
$^{15}N_5$-ALII-dA	518	402	40	22
$^{15}N_5$-8-oxo-dG	289	173	20	13

(B) AA-I and AA-II analysis

LC gradient

Time, min	Flow rate, mL/min	%A	%B
0.00	0.35	98.0	2.0
1.00	0.35	70.0	30.0
4.00	0.35	30.0	70.0
5.00	0.35	0.0	100.0
8.00	0.35	0.0	100.0
8.10	0.35	98.0	2.0
10.60	0.35	98.0	2.0

A: 0.1% ammonium acetate in water; B: acetonitrile

MS source parameter

Capillary Voltage, kV	1.5
Source Temperature, ^{o}C	150
Desolvation Temperature, ^{o}C	500
Cone gas flow, L/h	20
Desolvation gas flow, L/h	800

MS compound parameter

	**Parent ion, *m/z***	**Daughter ion, *m/z***	**Cone voltage, V**	**Collision energy, eV**
AA-I	359	324	8	8
	359	298	8	12
AA-II	329	294	12	8
	329	268	12	10
benz[*cd*]indol-2(1*H*)-one	170	115	25	38

(C) GSH and GSSG analysis

LC gradient

Time, min	Flow rate, mL/min	%A	%B
0.00	0.40	99.0	1.0
2.00	0.40	99.0	1.0
8.00	0.40	0.0	100.0
10.00	0.40	0.0	100.0
10.10	0.40	99.0	1.0
12.00	0.40	99.0	1.0

A: 0.2% acetic acid in water; B: acetonitrile

MS source parameter

Capillary Voltage, kV	0.4
Source Temperature, ^{o}C	150
Desolvation Temperature, ^{o}C	500
Cone gas flow, L/h	20
Desolvation gas flow, L/h	800

MS compound parameter

	**Parent ion, *m/z***	**Daughter ion, *m/z***	**Cone voltage, V**	**Collision energy, eV**
GSH	308	179	25	15
	308	76	30	25
GSSG	613	484	45	20
	613	355	45	25
[$^{13}C_2$, ^{15}N]-GSH	311	182	25	15
	311	79	30	25
[$^{13}C_4$, $^{15}N_2$]-GSSG	619	490	45	20
	619	361	45	25

(D) AL-I and AL-II analysis

LC gradient

Time, min	Flow rate, mL/min	%A	%B
0.00	0.30	90.0	10.0
1.00	0.30	30.0	70.0
7.00	0.30	55.0	45.0
10.00	0.30	40.0	60.0
10.10	0.30	100.0	0.0
13.50	0.30	100.0	0.0

A: 0.2% acetic acid in water; B: acetonitrile

Excitation wavelength: 254 nm; Emission wavelength: 451 nm

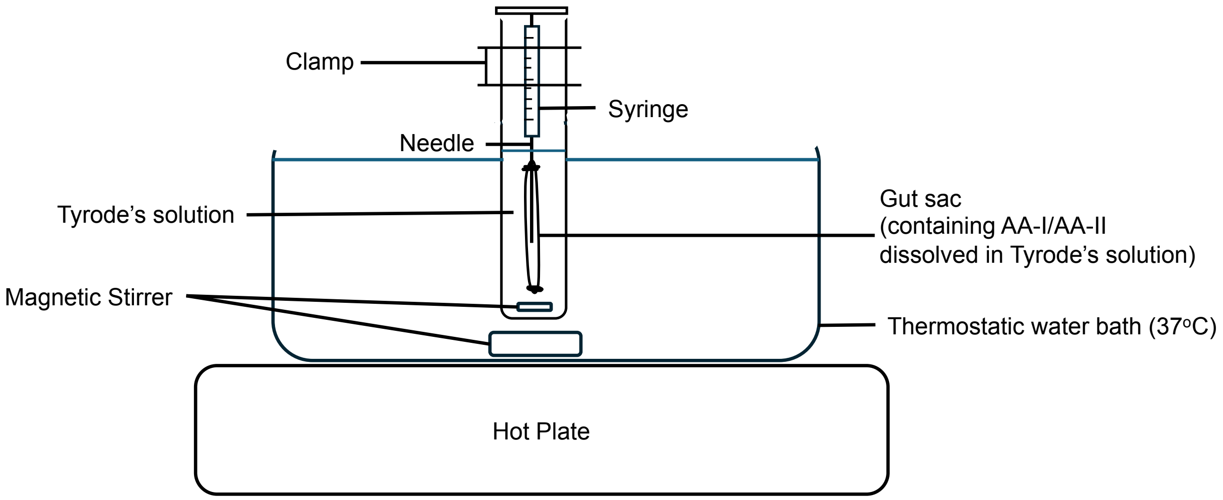

Figure S1. Schematic illustration of non-everted gut sac study set-up used in this study.

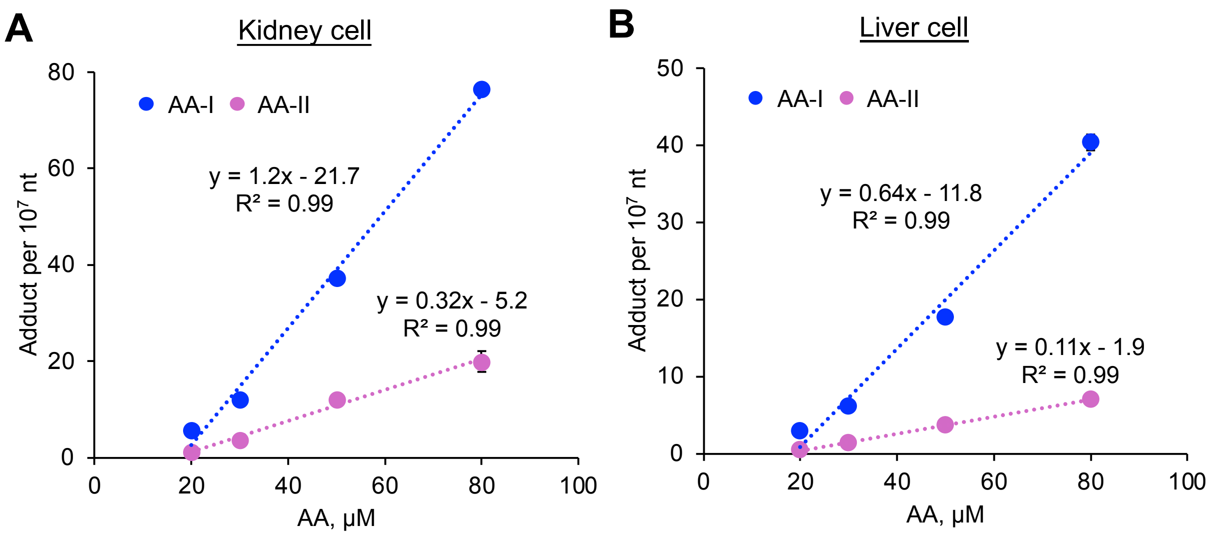

Figure S2. DNA adduct formation in cultured kidney (A) and liver (B) cells that were exposed to different concentrations of AA-I or AA-II for 48 h. The data represent means ± SD for three independent experiments.

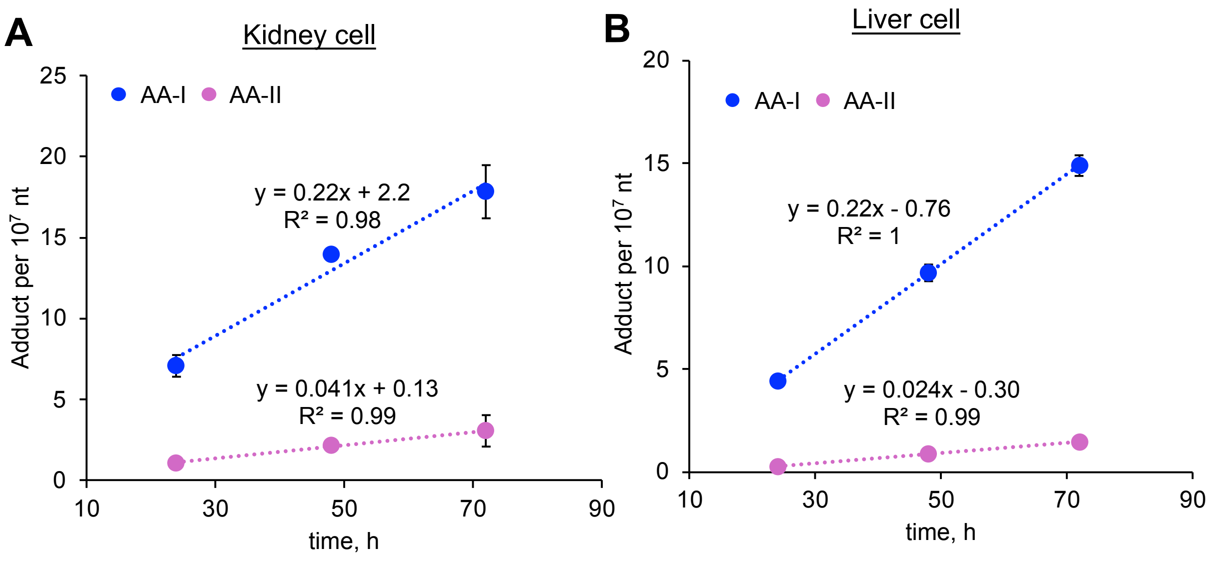

Figure S3. Exposure-time dependent formation of AA-DNA adduct (ALI-dA for AA-I; ALII-dA for AA-II) in cultured kidney (A) and liver (B) cells exposed to of 30 μM of AA-I and AA-II for varying duration. The data represent means ± SD for three independent experiments.

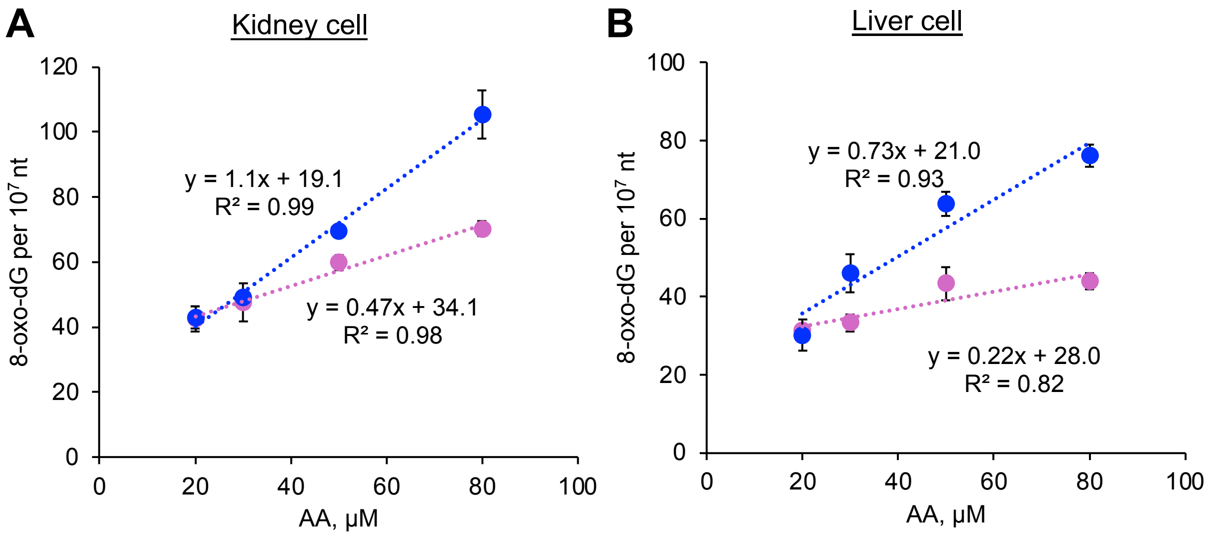

Figure S4. Level of 8-oxo-dG in cultured kidney (A) and liver (B) cells exposed to different concentrations of AA-I or AA-II for 48 h. The data represent means ± SD for three independent experiments.

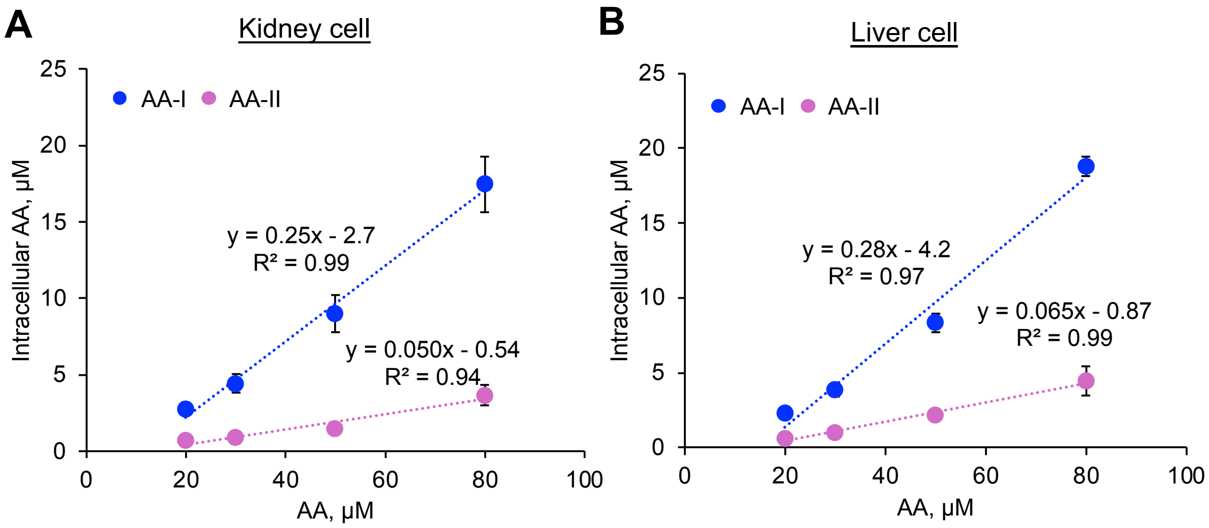

Figure S5. Concentrations of AA-I and AA-II in intracellular fluid of cultured kidney (A) and liver (B) cells exposed to different concentrations of AA-I or AA-II for 48 h. The data represent means ± SD for three independent experiments.

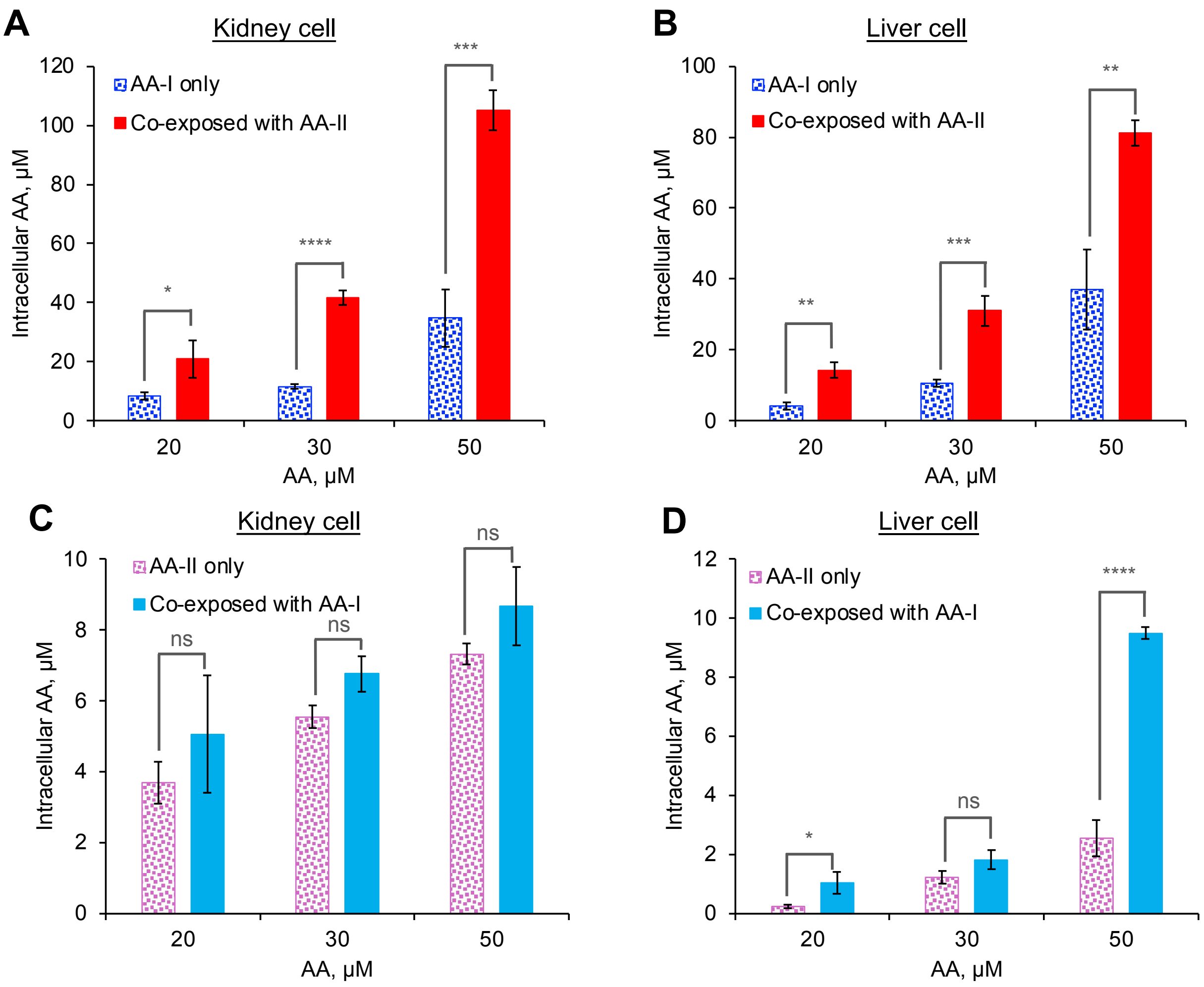

Figure S6. Concentrations of AA-I (A and B) and AA-II (C and D) in intracellular fluid of cultured kidney (A and C) and liver (B and D) cells co-exposed to different concentrations of AA-I and AA-II for 48 h. The data represent means ± SD for three independent experiments.

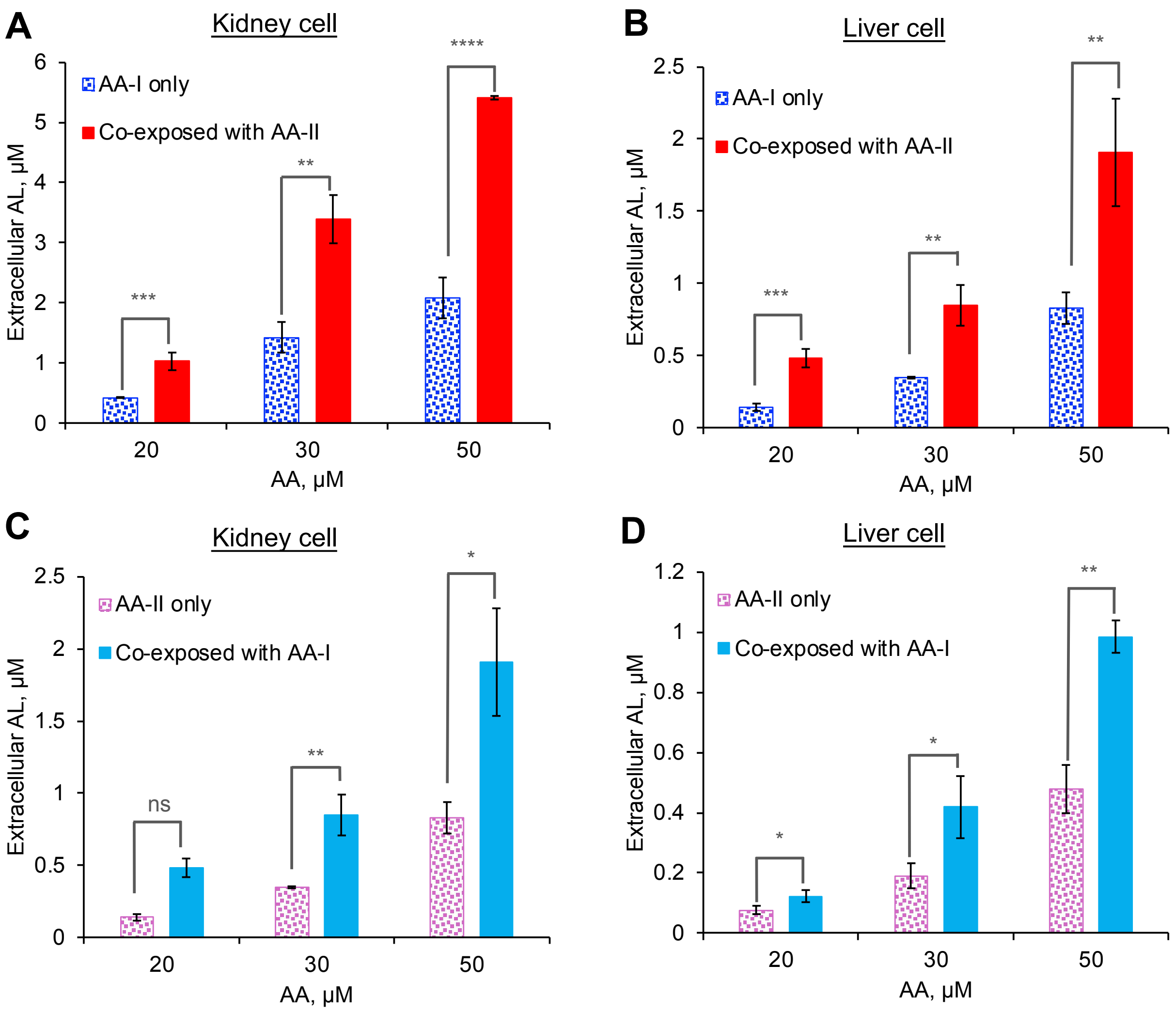

Figure S7. Concentrations of AL-I (A and B) and AL-II (C and D) in cell culture medium of cultured kidney (A and C) and liver (B and D) cells co-exposed to different concentrations of AA-I and AA-II for 48 h. The data represent means ± SD for three independent experiments.

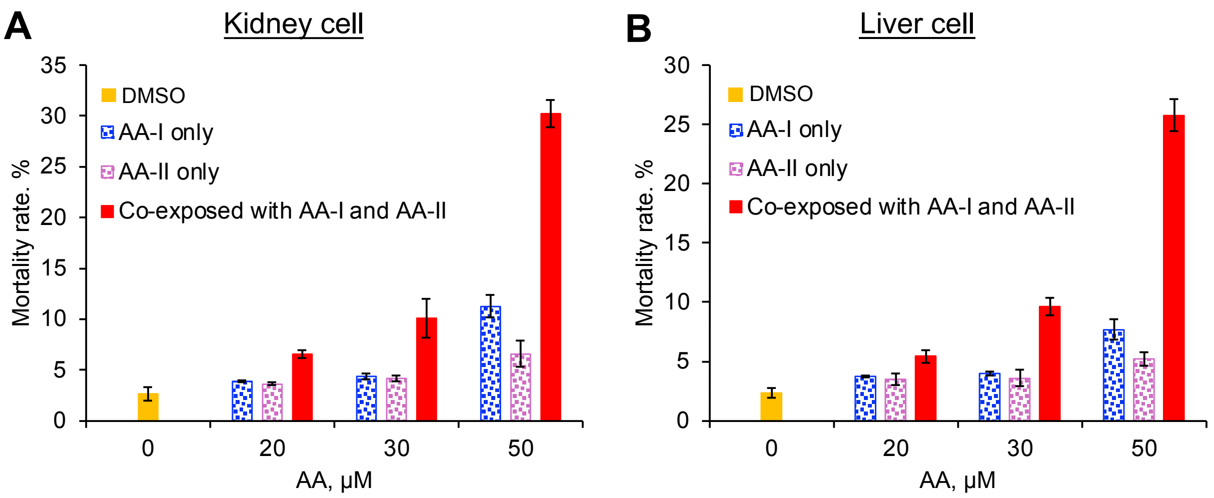

Figure S8. Cell mortality rates in cultured kidney (A) and liver (B) cells that were co-exposed to different concentrations of AA-I and AA-II for 48 h. The data represent means ± SD for three independent experiments.
